# Acetylenic Replacement of Albicidin's Methacrylamide Residue Circumvents Detrimental *E*/*Z* Photoisomerization and Preserves Antibacterial Activity

**DOI:** 10.1002/chem.202100523

**Published:** 2021-05-21

**Authors:** Iraj Behroz, Leonardo Kleebauer, Kay Hommernick, Maria Seidel, Stefan Grätz, Andi Mainz, John B. Weston, Roderich D. Süssmuth

**Affiliations:** ^1^ Institut für Organische Chemie Technische Universität Berlin Straße des 17. Juni 124 10623 Berlin Germany

**Keywords:** antibiotics, biological activity, drug discovery, medicinal chemistry, structure-activity relationships

## Abstract

The natural product albicidin is a highly potent inhibitor of bacterial DNA gyrase. Its outstanding activity, particularly against Gram‐negative pathogens, qualifies it as a promising lead structure in the search for new antibacterial drugs. However, as we show here, the N‐terminal cinnamoyl moiety of albicidin is susceptible to photochemical *E*/*Z* isomerization. Moreover, the newly formed *Z* isomer exhibits significantly reduced antibacterial activity, which hampers the development and biological evaluation of albicidin and potent derivatives thereof. Hence, we synthesized 13 different variants of albicidin in which the vulnerable *para*‐coumaric acid moiety was replaced; this yielded photostable analogues. Biological activity assays revealed that diaryl alkyne analogues exhibited virtually undiminished antibacterial efficacy. This promising scaffold will therefore serve as a blueprint for the design of a potent albicidin‐based drug.

## Graphical abstract

**Lead optimization**: The cinnamoyl residue of the antibiotic albicidin is shown to readily undergo UV‐mediated *E*/*Z* isomerization, thereby converting from the highly bioactive *E* form found in the natural product to the significantly less bioactive *Z* form. Acetylenic replacement of the susceptible methylacrylamide moiety affords a set of photochemically stable albicidin derivatives with virtually undiminished antibacterial activity.

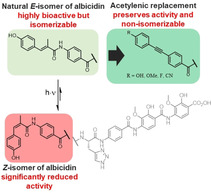

## Introduction

The global spread of antimicrobial resistance (AMR) increasingly renders medications used to treat life‐threatening infections ineffective and thus poses an imminent danger to millions of people.[^1^, ^2^] Even the strongest weapons in our therapeutic arsenal, such as polymyxins and carbapenems, are facing limitations due to the incessant emergence of pan‐resistant pathogenic bacteria.[^3^, ^4^] Due to their highly restrictive outer‐membrane permeability, Gram‐negative microorganisms are of particular concern.^[5]^ The most commonly used therapeutics, such as cephalosporins and quinolones, derive from known antibiotic classes that are remnants of the “golden era” of antibiotic discovery (the 1940s to 1960s). However, the development of improved analogues does not suffice anymore to keep pace with the spread of resistance. There is an urgent need for truly unique chemical scaffolds with unprecedented mechanisms of action.^[6]^ The clinical pipeline for first‐in‐class antibiotics is running dry because of, among other things, increasingly stringent regulatory barriers and major companies abandoning antibiotic research for the more lucrative field of chronic diseases.^[7]^

**Figure 1:**
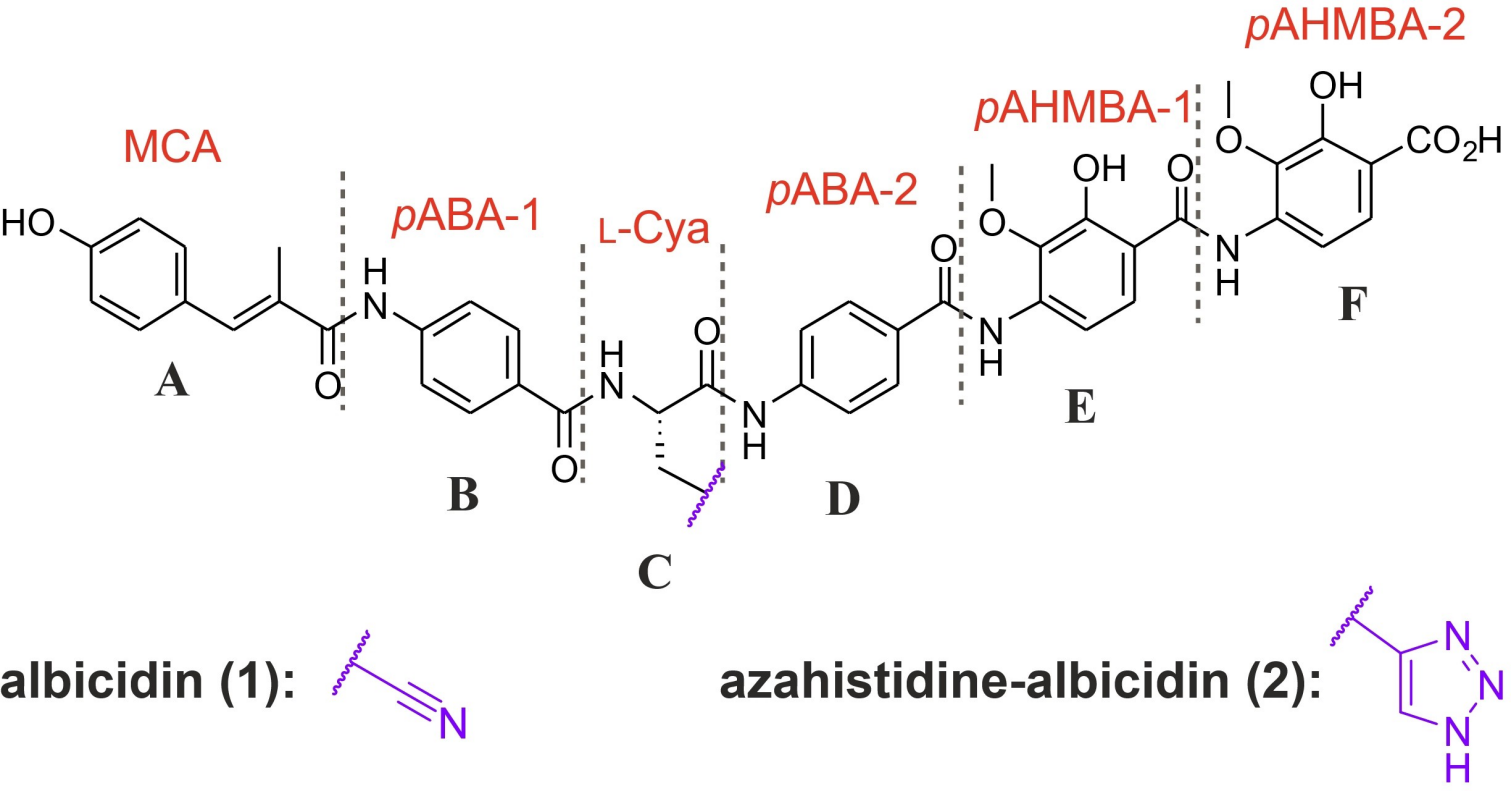
Structures of albicidin (**1**) and azahistidine‐albicidin (**2**). The individual building blocks A to F are labeled. Abbreviations for the systematic names of the individual fragments are highlighted in red.

First reported in 1985, the natural product albicidin (**1**, Figure 1) is a potential broad‐spectrum antibiotic with outstanding antibacterial activity against Gram‐positive and particularly Gram‐negative microorganisms in the nanomolar range.^[8]^ It effectively interferes with DNA replication, transcription, and gene regulation by inhibiting the supercoiling activity of bacterial DNA gyrase (topoisomerase II) with a half‐maximum inhibitory concentration (IC_50_) of 40 nm.^[9]^ This value is broadly similar to that of other important gyrase inhibitors, such as quinolones and coumarins.

However, albicidin's unusual structure and unique mode of action qualifies it as an auspicious lead structure in the search for a new antibacterial drug.^[10]^ Originally produced in low yields by the slow‐growing, plant‐pathogenic bacterium *Xanthomonas albilineans*, heterologous production by *Xanthomonas axonopodis* pv. *vesicatoria* has provided large‐enough quantities of albicidin, which enabled us to unveil its chemical structure^[11]^ and subsequently to establish its total synthesis.^[12]^ The phytotoxin **1** is produced by a complex polyketide synthase/nonribosomal peptide synthetase (PKS‐NRPS) biosynthetic machinery.^[13]^ As depicted in Figure 1, albicidin's oligoaromatic structure is composed of an unusual β‐cyano‐l‐alanine at its center (l‐Cya, building block C) flanked by two *p*‐aminobenzoic acids (*p*ABA‐1 and *p*ABA‐2, building blocks B and D). The C‐terminal dipeptide consists of two *para*‐amino‐2‐hydroxy‐3‐methoxybenzoic acids (*p*AHMBA‐1 and *p*AHMBA‐2, building blocks E and F).^[10]^ Finally, the N terminus consists of a methyl coumaric acid (MCA, building block A). The structurally related cystobactamids^[14]^ and coralmycins^[15]^ represent another group of oligoaromatic gyrase inhibitors. Their structures were elucidated almost in parallel to that of albicidin and had to be revised recently.[^16^, ^17^] Main structural differences include a *para*‐nitrobenzoic acid instead of MCA as building block A, a β‐methoxyasparagine in place of β‐cyanoalanine as building block C and *iso*‐propoxy groups instead of methoxy groups as decoration of building blocks E and F. The most important resistance mechanisms that have been discovered for albicidin include mutations in the *tsx* gene (nucleoside transporter) which block antibiotic uptake,[^18^, ^19^] enzymatic hydrolysis of the peptide by the endopeptidase AlbD from *Pantoea dispersa*,[^11^, ^20^] and nonenzymatic trapping of the molecule by the binding protein AlbA from *Klebsiella oxytoca*.[^21^, ^22^]

The scalable and diversifiable strategy employed in the total synthesis of albicidin has enabled the preparation of numerous synthetic and natural derivatives.[^12^, ^23^] In addition to variations of the N‐terminal cinnamoyl residue,^[24]^ initial structure‐activity relationship (SAR) studies have focused on the incorporation of various α‐amino acids as building block C,^[25]^ during which azahistidine‐albicidin (**2**, Figure 1) was identified as a more water‐soluble, new lead structure for further analogue synthesis. Recently, we reported an extensive SAR study for the C‐terminal dipeptide E−F,^[26]^ which gave valuable insights into the role of the substitution pattern of the aromatic rings and revealed a triazole amide‐bond isostere between building blocks D and E as a viable structural motif to overcome resistance by AlbD.^[11]^

Previous studies on albicidin's N‐terminal acyl group have demonstrated that antibacterial activity can be retained upon exchange of the hydroxy group in the *para* position of the aromatic ring.^[24]^ However, every derivative with considerable activity reported thus far contained an *E*‐configured cinnamoyl residue and was connected to the adjacent *p*ABA unit through an amide‐bond linker. In the past, cinnamates and their hydroxy derivatives, including *para*‐coumaric acid, caffeic acid, ferulic acid, and sinapic acid, have been shown to rapidly undergo light‐induced *E*/*Z* isomerization and dimerization, for example, in plant cell walls. The former two are known to isomerize from the *E* form to the *Z* form, and to a lesser degree in the opposite direction, to form an equilibrium mixture in which the *E* isomer is the main component.[^27^, ^28^, ^29^, ^30^] The photoisomerization of cinnamides is highly dependent on both N‐alkylation and aromatic substitution.[^31^, ^32^] In 1985, Birch and Patil had observed that albicidin gradually converted into another compound when stored in methanol at room temperature. This compound, which eluted before the natural product during HPLC analysis, exhibited diminished antibacterial activity.^[8]^ However, with the structure of albicidin being unknown at that time, the nature of the conversion also remained concealed. Similarly, in the past we have observed a shoulder in the UV chromatogram of albicidin and some of its derivatives during purification by preparative HPLC. Knowing today that the molecule contains several aryl amides as well as a cinnamoyl residue, we considered both atropisomerism^[33]^ and *E*/*Z* isomerism as potential causes for the conversion. In this study we show that albicidin indeed undergoes UV‐mediated conversion from the *E* to the *Z* form. More importantly, we also provide evidence that the *Z* isomer represents the less bioactive form. Therefore, the inherent susceptibility of the cinnamate to photochemical *E*/*Z* isomerization constitutes a major drawback and necessitates laborious precautionary measures during the synthesis, biological assessment, and storage of albicidin and respective analogues.

As a possible alternative to the methylacrylamide moiety we considered an acetylenic linker connecting the aromatic building blocks A and B. The use of the ethynyl group in pharmacologically active compounds dates to as early as 1961 when the syntheses of analgesics of the prodine‐type containing ethynyl or substituted ethynyl groups in place of the 4‐phenyl residue were reported.^[34]^ Ever since the acetylene group has been utilized as a potency enhancer, reactive warhead, nonpolar linear spacer, and nonclassical bioisostere. In the latter case it has served as a replacement for cyano, chloro, iodo, ethylene, carbonyl, ethyl, phenyl, cyclopropyl, and carboxamide groups.^[35]^ Among the few examples for FDA‐approved drugs bearing internal or terminal alkynes are the HIV‐1 reverse transcriptase inhibitor efavirenz (Sustiva®, Bristol‐Myers Squibb)[^36^, ^37^] and alkyne‐containing steroidal drugs. Concerning toxicity, the species‐dependent metabolism of efavirenz was shown to produce nephrotoxic glutathione conjugates in rats‐but not in humans[^38^, ^39^]‐while the steroidal drugs danazol^[40]^ and 17*α*‐ethynylestradiol^[41]^ are associated with a mechanism‐based inactivation of CYP450 in the liver. Therefore, the preclinical assessment of alkyne‐containing drug candidates requires the evaluation of metabolic pathways across CYP450 isoforms as well as the screening for reactive metabolites. Because of the knowledge and application of alkynes in medicinal chemistry, we were interested in assessing their applicability for structural variations of the antibacterial albicidin.

## Results and Discussion

**Figure 2:**
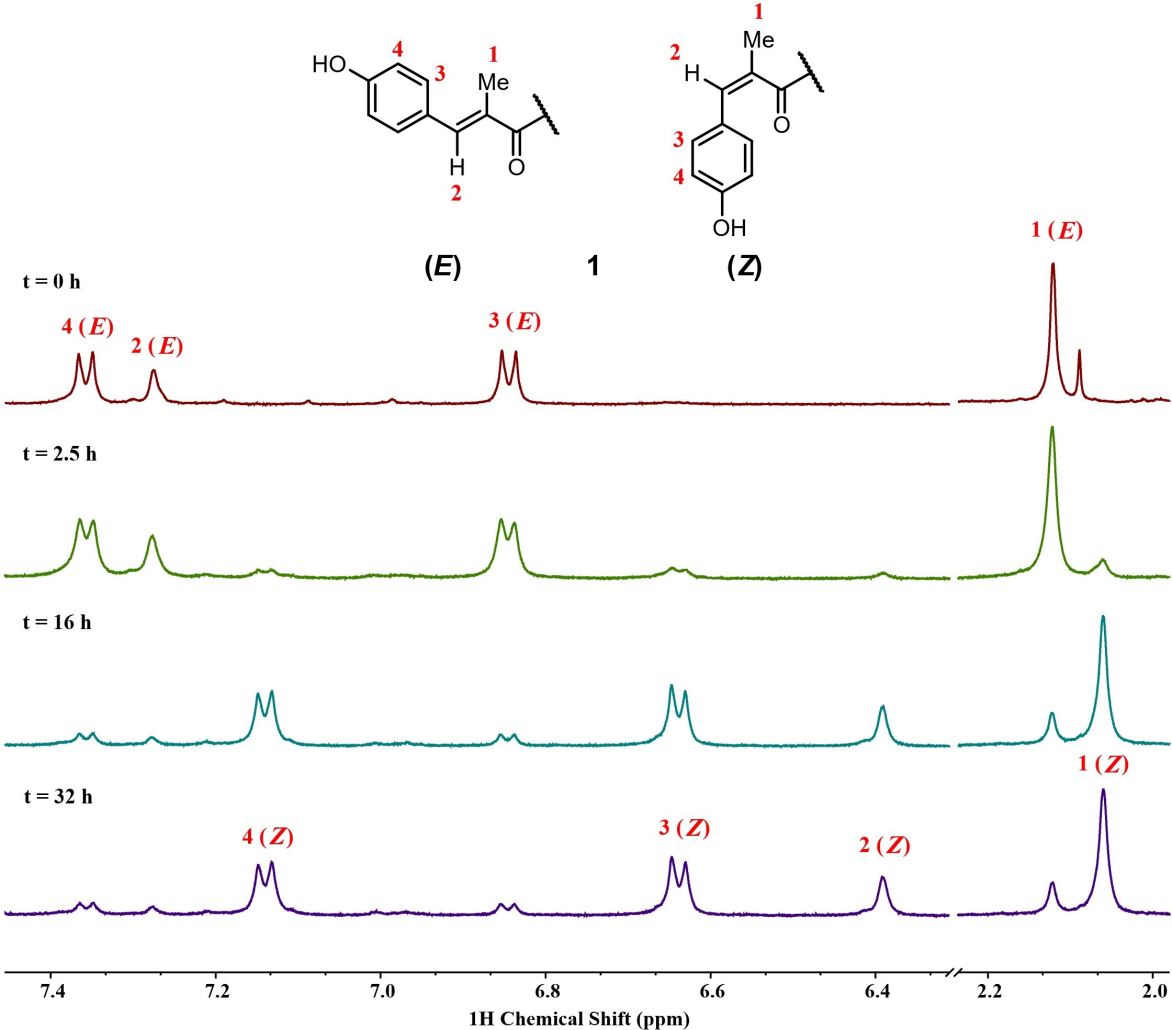
Photochemical *E*/*Z* isomerization of albicidin's cinnamoyl residue (building block A) monitored by ^1^H NMR spectroscopy over 32 h. The spectral regions of the aromatic/vinylic (*left*) and methyl protons (*right*) are shown. The two resonance sets for the *E* and *Z* isomers are labeled accordingly.

To demonstrate the photochemical (*E*)‐(*Z*)‐isomerization for the cinnamoyl residue of albicidin, a solution of freshly prepared albicidin (**1**) in [D_6_]DMSO was exposed to UV light (*λ*=366 nm) in a time‐dependent manner and monitored by ^1^H NMR spectroscopy (Figure 2). After UV exposure, we observed a second resonance set mainly for signals arising from residues MCA and *p*ABA‐1. The new signals for the methyl, vinyl and aromatic protons of MCA were clearly shifted up‐field. To provide evidence that the second resonance set arose from the *Z* isomer, we performed 2D nuclear Overhauser enhancement (NOE) measurements, which unambiguously confirmed the expected atomic distances in the two isomeric states (Figure S1 in the Supporting Information). While only a small portion of the initial *E* state *(t*=0 h) was converted after *t*=2.5 h, an almost complete conversion to the *Z* isomer was detected after *t*=16 h. At this point, the equilibrium state had already been reached (ca. 85 % *Z* form) and no further conversion was observed at *t*=32 h (Figure 2). Interestingly, we found that by replacing the *para*‐hydroxy group with an electron‐withdrawing fluorine, the conversion rate was decreased. After 32 h, however, the equilibrium had also been reached and approximately 65 % of the fluoro analogue adopted the *Z*‐isomeric form (Figure S2). It is important to note that significant accumulation of the (*Z*)‐isomer also occurred when albicidin samples were exposed to sunlight (ca. 50 and 80 % *Z* after 32 and 160 h daylight, respectively) underlining the vulnerability of **1** during routine handling. These findings became even more relevant when we determined minimal inhibitory concentrations (MICs) against a panel of bacteria (Table S1). Depending on the bacterial strain, the *Z* isomer exhibited a five‐ to tenfold decline in antibacterial activity compared to the well‐characterized *E* isomer of albicidin. Accordingly, the *Z* isomer was incapable of inhibiting DNA gyrase when provided at the IC_50_ value (45 nm) of the potent *E* isomer (Figure S3). The fact, that the albicidin producer *X. albilineans* inhabits the xylem of sugar cane, which is exposed to sunlight, could severely impact the stability of albicidin. Given these structural and functional differences between the isomeric states that complicate SAR studies, we envisaged a photochemically more robust scaffold of albicidin without compromising its biological activity.

**Figure 3:**
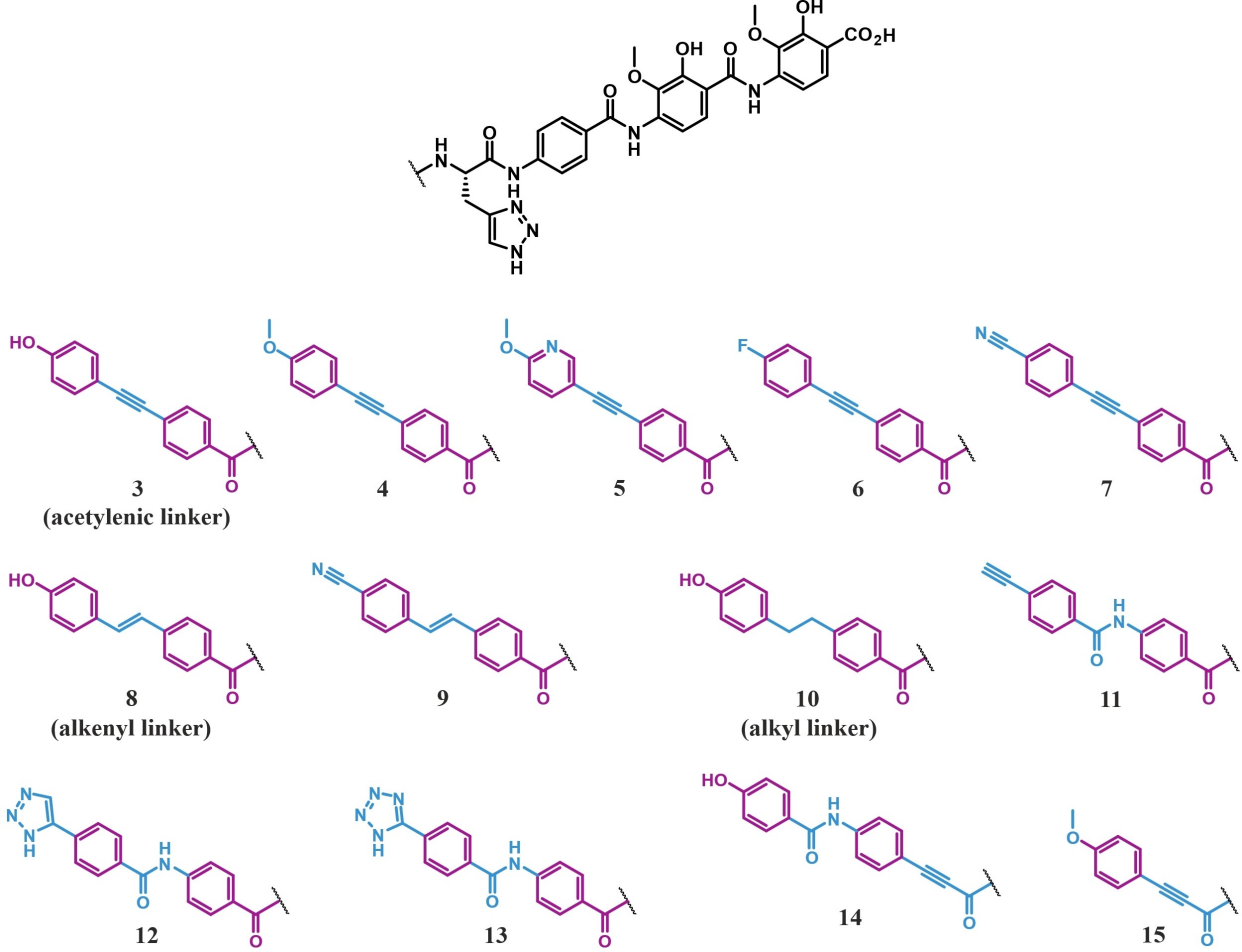
Structures of albicidin derivatives **3**–**15** with variations of the N‐terminal A−B building blocks and their connections. Unaltered structural features of albicidin are highlighted in purple, and all deviations from the template structure are highlighted in blue.

In our initial rationale we envisioned a synthetically feasible structural modification that could be considered a surrogate for *trans*‐configured amide bonds and could mimic the planarity and rigidity of albicidin's natural A‐B building block while increasing its photochemical stability. We hoped that a direct acetylenic linker between the two aromatic rings would be a viable replacement. Hence, we sought to replace the methacrylamide moiety between the cinnamate and the *p*ABA (building block B) with an alkyne, thus replacing the cinnamoyl‐*p*ABA with diarylalkyne carboxylates. We first prepared a set of five derivatives with varying substituents in the *para*‐position of the aromatic ring (Figure 3, compounds **3**–**7**). Based on previous findings,^[24]^ we included the lipophilic methoxy and fluoro groups in addition to the “hydro‐neutral” cyano group and the hydrophilic hydroxy group.^[42]^ We also included the methoxypyridine **5** to test the effect of a heterocyclic replacement in the aromatic ring adjacent to the *para*‐substituent on the biological activity of these derivatives.

**Scheme 1:**
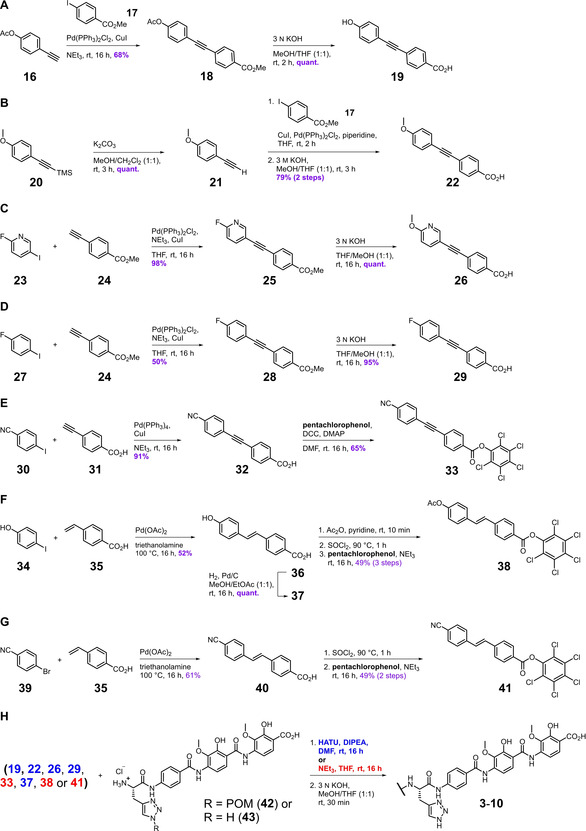
Synthetic pathways for the preparation of albicidin derivatives **3**–**10**. A)–G) Preparation of N‐terminal diaryl carboxylates with different linkers. H) Assembly of final albicidin derivatives **3**–**10** by coupling the diaryl carboxylates to tetrapeptides **42** and **43**. No coupling reagent was used for the reaction of activated PCP‐esters (red). All other A−B building blocks were coupled in the presence of HATU (blue). POM: pivaloyloxymethyl.

The acetylenic A−B building blocks were prepared from commercially available aryl alkynes and aryl halides employing well‐established palladium‐mediated cross‐coupling chemistry (Scheme 1A–E).[^43^, ^44^] The deprotected methoxypyridine **26** was prepared from the fluoropyridine precursor **25** through a nucleophilic aromatic substitution reaction using aqueous potassium hydroxide (Scheme 1C). The diaryl alkyne carboxylates **19**, **22**, **26** and **29** were activated by HATU before coupling to the recently reported and preformed tetrapeptide **42** (Scheme 1H).^[26]^ To prepare analogue **7**, compound **32** (Scheme 1E) was activated by forming the corresponding pentachlorophenol (PCP) ester **33** and coupled to the previously side‐chain POM‐deprotected tetrapeptide **43**. This approach obviated the need for the final deprotection step using aqueous potassium hydroxide and thus preserved the nitrile. To evaluate their antibacterial activity, MIC values for the fluoroquinolone ciprofloxacin (CIP), albicidin (**1**), azahistidine‐albicidin (**2**) and the newly synthesized derivatives **3**–**10** were determined for a panel of six different bacterial strains (Table 1). Likewise, a DNA gyrase supercoiling assay was performed to determine the ability of these compounds to inhibit the molecular target of albicidin. Notably, we found that the introduction of the acetylenic linker did not impair the antibacterial activity of the new compounds in the cell‐based assay at all. Rather, all tested derivatives remained highly active and produced MIC values in the range of albicidin (**1**). Cyano analogue **7** turned out to be the most active *para* substituent, showing low MIC values similar to those of CIP and azahistidine‐albicidin (**2**). Direct comparison of methoxy variant **4** and methoxypyridine variant **5** revealed that heterocyclic replacement of the aromatic ring did not have any effect on the antibacterial activity of the compound.

**Table 1:** MIC values^[a]^ for ciprofloxacin (CIP), albicidins **1** and **2**, and analogues **3**–**10** against a panel of six bacterial strains (BS).^[b]^

Entry	MIC [μg mL^−1^]					
	*E. c*. 1116	*E. c*. 25113	*S. t*. 100	*B. s*. 10	*M. l*. 1790	*M. p*. 750
CIP	0.016	≤0.016	≤0.016	0.125	1.0	0.25
**1**	0.063	0.063	0.016	0.25	2.0	2.0
**2**	0.016	n.d.^[c]^	≤0.016	0.125	0.5	1.0
**3**	0.063	0.125	0.031	2.0	2.0	1.0
**4**	0.063	0.125	≤0.016	0.25	0.5	1.0
**5**	0.063	0.063	≤0.016	0.25	0.5	1.0
**6**	0.031	0.031	0.016	0.25	0.25	0.5
**7**	≤0.016	0.031	≤0.016	0.25	0.25	1.0
**8**	0.5	0.25	0.125	0.5	1.0	4.0
**9**	0.031	0.063	≤0.016	0.063	0.031	1.0
**10**	0.5	0.25	0.125	2.0	2.0	4.0

[a] See the Supporting Information for detailed procedure. [b] *E. c*. 1116 (*E. coli* [DSM 1116], *E. c*. 25113 (*E. coli* [BW 25113]), *S. t*. 100 (*Salmonella typhimurium* [TA 100]), *B. s*. 10 (*B. subtilis* [DSM 10]), *M. l*. 1790 (*M. luteus* [DSM 1790]), *M. p. 750* (*M. phlei* [DSM 750]). [c] Not determined.

After demonstrating the viability of the diaryl alkyne motif as an alternative A−B building block, we decided to prepare the corresponding *trans*‐stilbene analogues **8** and **9** of variants **3** and **7** as well. Alkenes are prominent peptide‐bond isosteres because the C=C double bond closely mimics the C−N bond geometrically, while the electronic properties strongly differ from each other.[^45^, ^46^] First, the commercially available styrene **35** was coupled to the aryl halides **34** and **39** through a Heck reaction to produce stilbenes **36** and **40**, respectively (Scheme 1F and G).^[47]^ The final assembly of compounds **8** and **9** was achieved by employing the PCP‐ester strategy described above (Scheme 1H). The desired *trans*‐configuration of the double bond was confirmed for both stilbenes by the characteristic coupling constants of the olefinic protons in the ^1^H NMR spectra. To complete the picture, a fully saturated alkyl linker was incorporated into the molecule to give compound **10**. The required A−B building block **37** (structure not shown) was prepared from stilbene **36** employing catalytic hydrogenation (Scheme 1F).

As expected, the loss of planarity and increased flexibility of compound **10**, as compared to the acetylenic analogue **3**, led to a significant decrease of antibacterial activity of the molecule (Table 1). For instance, an eightfold increase of MIC values was determined for both tested *Escherichia coli* strains. However, except for slightly lower values for *Bacillus subtilis* and *Micrococcus luteus* strains, the stilbene analogue **8** exhibited similar potency to **10**. Interestingly, the antibacterial activity of stilbene **9** largely replicated the activity of corresponding alkyne **7**. The MIC values of compound **9** were slightly higher for *E. coli* and again slightly lower for *B. subtilis* and *M. luteus*. This appears to represent a general trend: by reducing the diaryl alkyne to the corresponding stilbene, the antibacterial activity decreases for Gram‐negative *E. coli* and increases for Gram‐positive *B. subtilis* and *M. luteus*. Intriguingly, only the two compounds bearing nitrile functionalities, i. e. analogues **7** and **10**, displayed considerable activity in the target‐based gyrase assay.

**Scheme 2:**
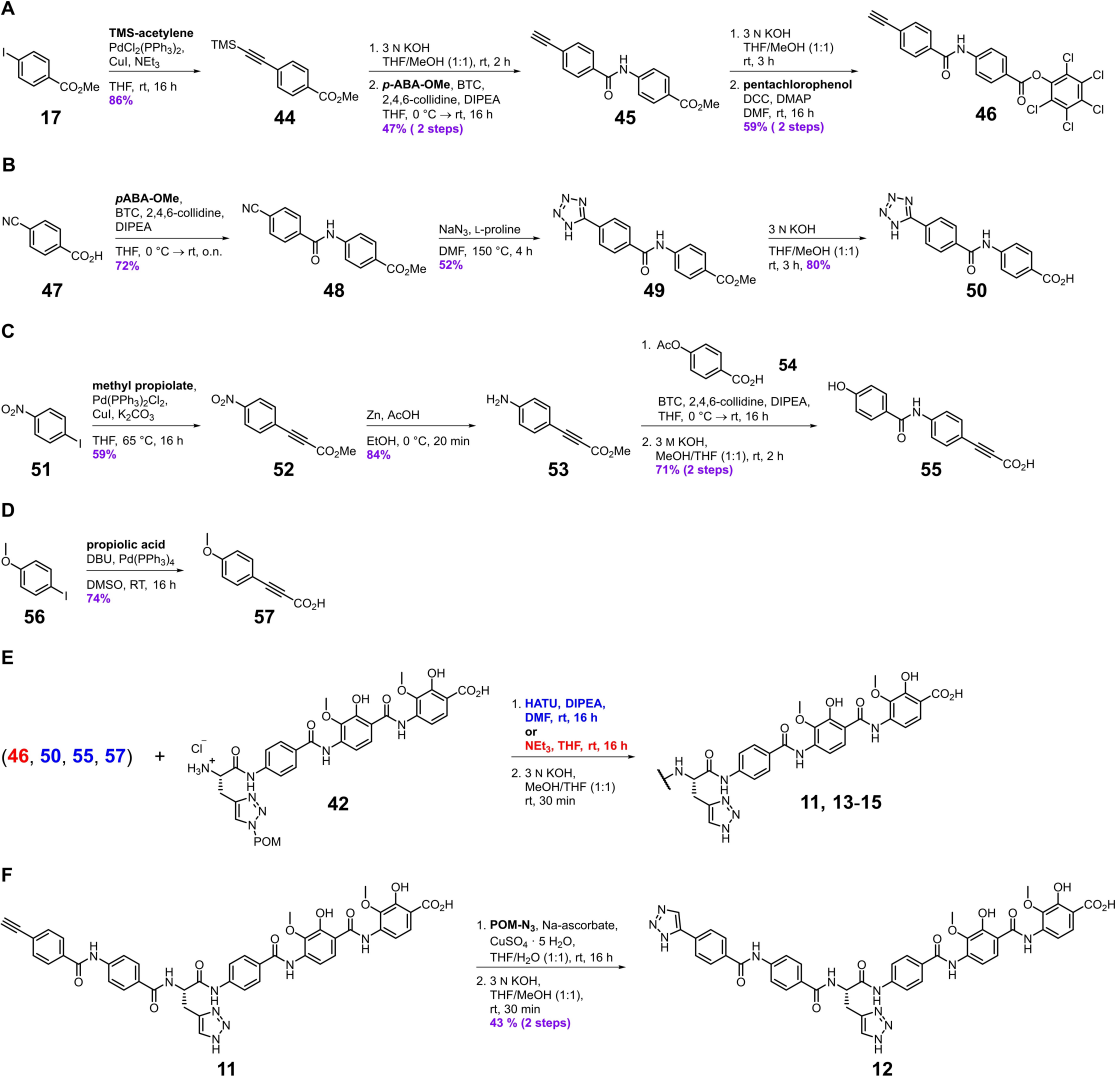
Synthetic pathways for the preparation of albicidin derivatives **11**–**15**. A)‐D) Preparation of modified N‐terminal building blocks **46**, **50**, **55**, and **57**. E) Assembly of final albicidin derivatives **11** and **13**–**15**. No coupling reagent was used for the reaction of PCP‐ester **46** (red). All other A−B building blocks were coupled in the presence of HATU (blue). F) Two‐step synthesis of triazole **12** from its acetylenic precursor **11**.

As the acetylenic compounds **3**–**7** performed very well in the inhibition assays, we were curious about the effect a terminal alkyne would have on antibacterial efficacy. For this purpose, we prepared variant **11** by coupling tetrapeptide **42** to the acetylenic benzamide **46**, the latter of which was prepared from aryl iodide **17** in a five‐step linear sequence initiated by a Sonogashira‐coupling to introduce the N‐terminal triple bond (Scheme 2A).^[48]^ Phenylacetylene **11** turned out to be highly active, and except for *B. subtilis* and *Mycobacterium phlei*, the MIC values have the same order of magnitude as for azahistidine‐albicidin (**2**) and CIP (Table 2). We then took advantage of the alkyne in place by carrying out a late‐stage copper‐catalyzed 1,3‐dipolar cycloaddition of compound **11** and POM‐azide,^[49]^ followed by removal of the protecting group to generate the triazole derivative **12** (Scheme 2F). Despite improved activities against *M. luteus* and *M. phlei*, the higher MIC values determined for the remaining panel of pathogens for compound **12**, as compared to its precursor alkyne **11**, suggests that the presumably increased polarity, size, and capacity for hydrogen‐bonding introduced to the molecule by the triazole ring have a deteriorating effect on the overall activity of **12**. Another aspect to be considered is the shift from a weakly to non‐acidic C−H bond of the alkyne to a weakly basic triazole. We then installed an acidic N‐terminal head group by replacing the triazole with a tetrazole, which is commonly utilized in medicinal chemistry as a bioisosteric replacement for carboxylic acids.^[50]^ The tetrazole‐containing derivative **13** was prepared from benzonitrile **47** in five steps, involving an l‐proline catalyzed [3+2]‐cycloaddition as the key step (Scheme 2B).^[51]^ Introduction of the negatively charged head group resulted in a 30‐fold decrease of activity against Gram‐negative *E. coli* and *S. typhimurium* strains, as well as at least an eightfold decrease against Gram‐positive *B. subtilis* and *M. luteus*.

**Table 2:** MIC values^[a]^ for ciprofloxacin (CIP), albicidins **1** and **2**, and analogues **11**−**15** against a panel of six bacterial strains (BS).^[b]^

Entry	MIC [μg mL^−1^]					
	*E. c*. 1116	*E. c*. 25113	*S. t*. 100	*B. s*. 10	*M. l*. 1790	*M. p*. 750
CIP	0.016	≤0.016	≤0.016	0.125	1.0	0.25
**1**	0.063	0.063	0.016	0.25	2.0	2.0
**2**	0.016	n.d.^[b]^	≤0.016	0.125	0.5	1.0
**11**	0.031	0.016	≤0.016	0.125	2.0	8.0
**12**	0.063	0.125	0.063	1.0	0.125	2.0
**13**	2.0	4.0	2.0	≥8.0	8.0	8.0
**14**	0.5	0.5	0.5	≥8.0	≥8.0	≥8.0
**15**	≥8.0	≥8.0	≥8.0	≥8.0	≥8.0	≥8.0

[a] See the Supporting Information for detailed procedure. [b] *E. c*. 1116 (*E. coli* [DSM 1116], *E. c*. 25113 (*E. coli* [BW 25113]), *S. t*. 100 (*S. typhimurium* [TA 100]), *B. s*. 10 (*B. subtilis* [DSM 10]), *M. l*. 1790 (*M. luteus* [DSM 1790]), *M. p. 750* (*M. phlei* [DSM 750]). [c] Not determined.

Previously serving as an N‐terminal head group (compound **11**) and as a direct linker between building blocks A and B (compounds **3**–**7**), the triple bond was relocated again to partake in the formation of an elongated hybrid alkyne–amide link between building blocks B and C (compounds **14** and **15**, Figure 3). The *para*‐hydroxybenzamide **14** and the truncated anisole variant **15** were both synthesized from commercially available aryl iodides employing the same synthetic strategies described above (Scheme 2C and D). As the microdilution assay revealed (Table 2), analogue **15** suffered from a complete loss of activity, which potentially stems from the unfavorable length of the molecule. Similarly, variant **14** displayed no activity against the Gram‐positive strains and a 30‐fold lower activity against *E. coli* and *S. typhimurium*.

**Figure 4:**
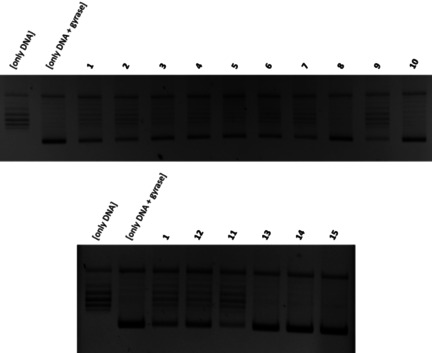
DNA gyrase inhibition assay for albicidin (1), azahistidine‐albicidin (2) and analogues **3**–**15**. The control experiment without enzyme and drug (left lane) shows relaxed DNA. Addition of DNA gyrase results in supercoiled DNA (second lane from left). All derivatives were tested at a concentration of 45 nM. See the Supporting Information for detailed procedure.

The results of the target‐based *E. coli* DNA gyrase assay are consistent with the results obtained from the cell‐based MIC assay (Figure 4). All acetylenic derivatives (**3**–**7**) inhibited the enzyme's activity as well as albicidin (**1**). Again, the cyano analogue **7** turned out to be the most potent one, exhibiting similar activity to azahistidine‐albicidin **2**. The partial loss of antibacterial activity observed for the reduced analogues **8** and **10** is also reflected in their diminished capacity to inhibit DNA gyrase. At the same time, cyano stilbene **9**, as well as phenylacetylene **11**, were demonstrated to be highly potent against the molecular target. Consequently, the triazole derivative **12** showed a decreased activity as compared to its acetylenic precursor. Finally, the tetrazole variant **13**, 4‐hydroxybenzamide **14**, and the truncated anisole **15** did not show any activity in this assay either.

## Conclusion

In summary, we have shown that the cinnamoyl group of albicidin is susceptible to *E*/*Z* photoisomerization and that its *Z* isomer is significantly less active. Hence, it would be preferable to substitute this functional group with alternative ones. To this end, we have synthesized 13 new albicidin derivatives with variations of the N‐terminal dipeptide to obtain photochemically stable but highly active albicidin analogues. We identified an acetylenic linker between building blocks A and B as a viable replacement for the methacrylamide moiety found in the natural product. Notably, albicidin's pronounced antibacterial activity was completely retained for diaryl nitrile **7** and only slightly diminished for the corresponding stilbene analogue **9**. These results are very promising, qualify the acetylenic group as a possible substitute in the original structure of albicidin, and further support the SAR‐guided search for an eligible clinical drug candidate.

## Experimental Section

**Final derivative 3**: HATU (53 mg, 140 μmol, 1.5 equiv) was added to a solution of diaryl alkyne 19 (29 mg, 121 μmol, 1.3 equiv) in anhyd. DMF (3 mL) and the resulting solution was stirred at room temperature for 15 min. A solution of tetrapeptide 42 (70 mg, 93 μmol, 1.0 equiv) and DIPEA (0.12 mL) in anhyd. DMF (1 mL) was added dropwise and the reaction mixture was stirred at room temperature for 16 h. All volatiles were removed *in vacuo* and the residue was taken up in a mixture of THF (1 mL) and MeOH (1 mL), and 3 n KOH_(aq.)_ (1 mL) was added to that solution dropwise. After 30 min of stirring, 3 n HCl_(aq.)_ (1.1 mL) was added and the resulting suspension was evaporated under reduced pressure. The crude material was dissolved in DMSO, centrifuged, and the supernatant purified by HPLC (PLRP−S, CH_3_CN in H_2_O). The title compound 3 (12 mg, 16 % over two steps) was obtained as a colorless solid. ^1^H NMR (500 MHz, [D_6_]DMSO): *δ*=11.52 (s, 1H), 11.17 (s, 1H), 10.52 (s, 1H), 9.99 (s, 1H), 9.66 (s, 1H), 8.92 (d, *J*=7.3 Hz, 1H), 8.06 (d, *J*=8.9 Hz, 1H), 7.98 (d, *J*=8.7 Hz, 2H), 7.91 (d, *J*=8.4 Hz, 2H), 7.75–7.84 (m, 3H), 7.56–7.64 (m, 4H), 7.41 (d, *J*=8.5 Hz, 2H), 6.82 (d, *J*=8.0 Hz, 2H), 4.89–4.99 (m, *J*=6.3 Hz, 1H), 3.92 (s, 3H), 3.79 (s, 3H), 3.21–3.41 (m, 2H). ^13^C NMR (126 MHz, [D_6_]DMSO): *δ*=133.7 (Ar), 131.4 (Ar), 129.1 (Ar), 128.3 (Ar), 126.1 (Ar), 119.2 (Ar), 116.3 (Ar), 115.3 (Ar), 110.7 (Ar), 60.6 (OMe), 61.0 (OMe), 54.8 (α‐C), 29.4 (β‐C). HRMS (ESI): *m/z* calcd for C_43_H_35_N_7_O_11_ [*M*+H]^+^ 826.2467; found 826.2454 (Δ*m*=−1.6 ppm), *t*_R_=8.38 min.

**Final derivative 4**: Synthetic protocol analogous to compound **3**. The title compound **4** was obtained as a colorless solid (4 mg, 9 % over two steps). ^1^H NMR (400 MHz, [D_6_]DMSO): *δ*=11.54 (s, 1H), 11.19 (s, 1H), 10.55 (s, 1H), 9.69 (s, 1H), 8.95 (d, *J*=7.8 Hz, 1H), 8.06 (d, *J*=9.0 Hz, 1H), 7.97 (d, *J*=8.8 Hz, 2H), 7.92 (d, *J*=8.5 Hz, 2H), 7.75–7.84 (m, 3H), 7.63 (d, *J*=8.5 Hz, 2H), 7.56–7.61 (m, 2H), 7.51–7.56 (m, 2H), 6.98–7.04 (m, 2H), 4.88–4.97 (m, 1H), 3.91 (s, 3H), 3.80 (s, 3H), 3.78 (s, 3H), 3.19–3.35 (m, *J*=5.5 Hz, 2H). ^13^C NMR (101 MHz, [D_6_]DMSO): *δ*=133.4 (Ar), 131.4 (Ar), 129.0 (Ar), 128.1 (Ar), 128.1 (Ar), 125.8 (Ar), 125.6 (Ar), 119.1 (Ar), 115.2 (Ar), 114.9 (Ar), 110.8 (Ar), 60.8 (OMe), 60.8 (OMe), 55.7 (OMe), 54.5 (α‐C), 27.6 (β‐C). HRMS (ESI): *m/z* calcd for C_44_H_37_N_7_O_11_ [*M*+H]^+^ 840.2624; found 840.2626 (Δ*m*=+0.2 ppm), *t*_R_=9.38 min.

**Final derivative 5**: Synthetic protocol analogous to compound **3**. The title compound **5** was obtained as a colorless solid (12 mg, 15 % over two steps). ^1^H NMR (400 MHz, [D_6_]DMSO): *δ*=11.56 (br. s., 1H), 11.20 (s, 1H), 10.56 (s, 1H), 9.69 (s, 1H), 8.98 (d, *J*=7.5 Hz, 1H), 8.44 (d, *J*=2.0 Hz, 1H), 8.06 (d, *J*=8.8 Hz, 1H), 7.87–8.01 (m, 5H), 7.76–7.84 (m, 3H), 7.63–7.72 (m, 3H), 7.60 (d, *J*=5.5 Hz, 1H), 7.58 (d, *J*=5.5 Hz, 1H), 6.91 (d, *J*=8.8 Hz, 1H), 4.88–4.99 (m, 1H), 3.91 (s, 3H), 3.90 (s, 3H), 3.78 (s, 3H), 3.20–3.37 (m, 2H). ^13^C NMR (101 MHz, [D_6_]DMSO): *δ*=150.4 (Ar), 142.0 (Ar), 131.5 (Ar), 128.7 (Ar), 126.0 (Ar), 125.9 (Ar), 119.2 (Ar), 115.2 (Ar), 111.2 (Ar), 110.9 (Ar), 60.8 (OMe), 54.7 (OMe), 54.3 (α‐C), 54.0 (OMe), 27.1 (β‐C). HRMS (ESI): *m/z* calcd for C_43_H_36_N_8_O_11_ [*M*+H]^+^ 841.2576; found 841.2584 (Δ*m*=+1.0 ppm), *t*_R_=8.63 min.

**Final derivative 6**: Synthetic protocol analogous to compound **3**. The title compound **6** was obtained as a colorless solid (25 mg, 32 % over two steps). ^1^H NMR (400 MHz, [D_6_]DMSO): *δ*=11.55 (s, 1H), 11.19 (s, 1H), 10.56 (s, 1H), 9.69 (s, 1H), 8.98 (d, *J*=7.5 Hz, 1H), 8.06 (d, *J*=9.0 Hz, 1H), 7.96 (dd, *J*=8.53, 15.6 Hz, 4H), 7.76–7.85 (m, 3H), 7.63–7.73 (m, 5H), 7.59 (dd, *J*=5.4, 8.9 Hz, 2H), 7.30 (t, *J*=8.9 Hz, 2H), 4.88–4.99 (m, 1H), 3.92 (s, 3H), 3.78 (s, 3H), 3.19–3.37 (m, 2H). ^13^C NMR (101 MHz, [D_6_]DMSO): *δ*=134.2 (Ar), 132.7 (Ar),129.4 (Ar),128.1 (Ar), 126.1 (Ar), 125.8 (Ar), 119.1 (Ar), 116.5 (Ar), 115.3 (Ar), 110.6 (Ar), 60.8 (OMe), 60.6 (OMe), 54.7 (α‐C), 27.6 (β‐C). HRMS (ESI): *m*/*z* calcd for C_43_H_34_FN_7_O_10_ [*M*+H]^+^ 828.2424; found 828.2427 (Δ*m*=+0.4 ppm) *t*_R_=9.04 min.

**Final derivative 7**: The tetrapeptide 43 (60 mg, 93 μmol, 1.0 equiv) and the active ester 33 (52 mg, 102 μmol, 1.1 equiv) were dissolved in a mixture of anhyd. DMF (5 mL) and triethylamine (0.10 mL, 744 μmol, 8.0 equiv). The mixture was stirred at room temperature for 16 h. All volatiles were removed *in vacuo* and the crude material was dissolved in DMSO and purified by HPLC (PLRP−S, CH_3_CN in H_2_O). The title compound 7 (5.0 mg, 6 %) was obtained as a colorless powder. ^1^H NMR (400 MHz, [D_6_]DMSO): *δ*=11.54 (s, 1H), 11.17 (s, 1H), 10.56 (s, 1H), 9.68 (s, 1H), 9.01 (d, *J*=8.0 Hz, 1H), 7.77–7.83 (m, 5H), 7.73 (d, *J*=8.5 Hz, 2H), 7.60 (d, *J*=3.5 Hz, 1H), 7.57 (d, *J*=3.8 Hz, 1H), 4.88–4.99 (m, 1H), 3.92 (s, 3H), 3.78 (s, 3H). ^13^C NMR (101 MHz, [D_6_]DMSO): *δ*=132.9 (Ar), 132.7 (Ar), 132.0 (Ar), 128.8 (Ar), 126.3 (Ar), 119.1 (Ar), 115.3 (Ar), 110.3 (Ar), 60.8 (OMe), 60.5 (OMe). HRMS (ESI): *m/z* calcd for C_44_H_34_N_8_O_10_ [*M*+H]^+^ 835.2471; found 835.2469 (Δ*m*=−0.2 ppm), *t*_R_=9.38 min.

**Final derivative 8**: The tetrapeptide 42 (0.16 g, 0.22 mmol, 1.0 equiv) and the active ester 38 (0.11 g, 0.21 mmol, 1.0 equiv) were dissolved in a mixture of anhyd. DMF (3 mL) and triethylamine (0.29 mL, 2.1 mmol, 10 equiv). The mixture was stirred at room temperature for 16 h. All volatiles were removed *in vacuo* and the residue was taken up in a mixture of THF (2 mL) and MeOH (2 mL), and 3 n KOH_(aq.)_ (2 mL) was added dropwise at 0 °C. The ice bath was removed and after 15 min of stirring 3 n HCl_(aq.)_ (2.1 mL) was added and the resulting suspension was concentrated under reduced pressure. The crude material was dissolved in DMSO, centrifuged, and the supernatant was purified by HPLC (PLRP−S, CH_3_CN in H_2_O). The title compound 8 (3.0 mg, 2 % over two steps) was obtained as a colorless powder. ^1^H NMR (500 MHz, [D_6_]DMSO): *δ*=10.82 (br s, 1H), 10.59 (s, 1H), 9.61 (s, 1H), 8.79–8.84 (m, 1H), 7.97 (d, *J*=9.0 Hz, 2H), 7.88 (d, *J*=8.4 Hz, 2H), 7.80 (br d, *J*=8.9 Hz, 3H), 7.66–7.70 (m, 1H), 7.60–7.66 (m, 2H), 7.55 (d, *J*=8.9 Hz, 1H), 7.41–7.51 (m, 3H), 7.28 (d, *J*=16.5 Hz, 1H), 7.07 (d, *J*=16.5 Hz, 1H), 6.77–6.84 (m, 2H), 4.90–4.96 (m, 1H), 3.86 (s, 3 H), 3.79 (s, 3 H). ^13^C NMR (126 MHz, [D_6_]DMSO): *δ*=130.7 (Ar‐C*H*), 128.9, 128.4, 128.2, 125.9, 125.2, 125.0, 124.7 (C*H*‐Ar), 124.4, 119.0 (Ar), 115.9 (Ar), 114.6 (Ar), 107.8 (Ar), 60.6 (OMe), 59.8 (OMe), 54.6 (α‐C), 27.5 (β‐C). HRMS (ESI): *m*/*z* calcd for C_43_H_37_N_7_O_11_ [*M*+H]^+^ 828.2624; found 828.2628 (Δ*m*=−0.4 ppm), *t*_R_=8.11 min.

**Final derivative 9**: Synthetic protocol analogous to compound **8**. The title compound **9** was obtained as a colorless solid (2.3 mg, 2 % over two steps). ^1^H NMR (500 MHz, [D_6_]DMSO): *δ*=11.54 (br s, 1H), 11.17 (s, 1H), 10.58 (s, 1H), 9.67 (s, 1H), 8.86–8.91 (m, 1H), 8.02–8.08 (m, 1H), 7.97 (d, *J*=8.7 Hz, 2H), 7.93 (d, *J*=8.1 Hz, 2H), 7.77–7.87 (m, 8H), 7.75 (d, *J*=8.4 Hz, 2H), 7.69 (br s, 1 H), 7.60–7.55 (m, 2H), 7.51 (d, *J*=13.9 Hz, 2H), 4.90–4.97 (m, 1H), 3.91 (s, 3H), 3.77–3.79 (m, 3H), 3.77–3.79 (m, 3 H). ^13^C NMR (126 MHz, [D_6_]DMSO): *δ*=133.1, 131.2 (Ar‐C*H*), 129.8 (C*H*‐Ar), 129.6 (Ar), 128.6 (Ar), 127.4 (Ar), 127.4 (Ar), 126.1 (Ar), 119.3 (Ar), 115.3 (Ar), 110.8 (Ar), 61.1 (OMe), 61.0 (OMe), 54.8 (α‐C), 27.9 (β‐C). HRMS (ESI): *m*/*z* calcd for C_44_H_36_N_8_O_10_ [*M*+H]^+^ 837.2627; found 837.2631 (Δ*m*=−0.4 ppm), *t*_R_=8.87 min.

**Final derivative 10**: Synthetic protocol analogous to compound **3**. The title compound **9** was obtained as a colorless solid (4.0 mg, 2 % over two steps). ^1^H NMR (400 MHz, [D_6_]DMSO): *δ*=11.52 (s, 1H), 11.16 (s, 1H), 10.49 (s, 1H), 9.65 (s, 1H), 9.01–9.22 (m, 1H), 8.68–8.75 (m, 1H), 8.05 (d, *J*=8.8 Hz, 1H), 7.93–8.00 (m, 2H), 7.73–7.82 (m, 5H), 7.63–7.70 (m, 1H), 7.54–7.63 (m, 2H), 7.29 (d, *J*=8.0 Hz, 2H), 6.99 (d, *J*=8.5 Hz, 2H), 6.65 (d, *J*=8.3 Hz, 2H), 4.85–4.95 (m, 1H), 3.92 (s, 3H), 3.78 (s, 3H), 3.06–3.14 (m, 2H), 2.84–2.91 (m, 2H), 2.74–2.81 (m, 2H). ^13^C NMR (101 MHz, [D_6_]DMSO): *δ*=129.8 (Ar), 129.4 (Ar), 119.2 (Ar), 127.9 (Ar), 128.8 (Ar), 115.6 (Ar), 60.5 (Ar), 60.9 (Ar), 46.1 (CH_2_), 37.4 (CH_2_), 36.1 (CH_2_). HRMS (ESI): *m/z* calcd for C_43_H_39_N_7_O_11_ [*M*+H]^+^ 830.2780; found 830.2780 (Δ*m*=0 ppm), *t*_R_=8.23 min.

**Final derivative 11**: Synthetic protocol analogous to compound **8**. The title compound **11** was obtained as a colorless solid (15 mg, 18 % over two steps). ^1^H NMR (400 MHz, [D_6_]DMSO): *δ*=11.54 (s, 1H), 11.19 (s, 1H), 10.55 (s, 1H), 10.54 (s, 1H), 9.69 (s, 1H), 8.76 (d, *J*=7.5 Hz, 1H), 8.06 (d, *J*=9.0 Hz, 1H), 7.98 (dd, *J*=6.7, 8.2 Hz, 4H), 7.85−7.94 (m, 4H), 7.76−7.84 (m, 3H), 7.62−7.72 (m, 3H), 7.59 (dd, *J*=4.9, 8.9 Hz, 2H), 4.88−4.98 (m, 1H), 4.44 (s, 1H), 3.92 (s, 3H), 3.78 (s, 3H), 3.18−3.38 (m, 2H). (^1^H,^13^C)‐HSQC NMR (400 MHz, [D_6_]DMSO): *δ*=132.1 (Ar), 129.0 (Ar), 128.7 (Ar), 125.9 (Ar), 126.0 (Ar), 119.7 (Ar), 119.2 (Ar), 115.0 (Ar), 110.6 (Ar), 83.7 (alkyne), 61.0 (OMe), 60.5 (OMe), 54.4 (α‐C), 27.7 (β‐C); HRMS (ESI): *m/z* calcd for C_44_H_36_N_8_O_11_ [*M*+H]^+^ 853.2576; found 853.2585 (Δ*m*=+1.1 ppm), *t*_R_=8.34 min.

**Final derivative 12**: To a solution of the alkyne derivative **11** (45 mg, 47 μmol, 1.0 equiv) and azidomethyl pivalate (5.1 μL, 47 μmol, 1.0 equiv) in a mixture of THF (2 mL) and H_2_O (2 mL) was added CuSO_4_ ⋅ 5 H_2_O (0.58 mg, 2.3 μmol, 0.05 equiv). After purging the solution with N_2_ for 5 min, sodium ascorbate (1.8 mg, 9.3 μmol, 0.20 equiv) was added and the reaction mixture was stirred at room temperature for 16 h. MeOH (2 mL) and 3 n KOH_(aq.)_ (2 mL) were added and the solution was stirred for another 15 min at room temperature. After adding 3 n HCl_(aq.)_ (2.1 mL), the resulting suspension was concentrated under reduced pressure. The crude material was dissolved in DMSO, centrifuged, and the supernatant was purified by HPLC (PLRP−S, CH_3_CN in H_2_O). The title compound 12 (15 mg, 36 % over two steps) was obtained as a colorless powder. ^1^H NMR (400 MHz, [D_6_]DMSO): *δ*=11.55 (s, 1H), 11.19 (s, 1H), 10.57 (s, 1H), 10.53 (s, 1H), 9.69 (s, 1H), 8.77 (d, *J*=7.3 Hz, 1H), 8.01−8.14 (m, 5H), 8.0 (d, *J*=8.8 Hz, 2H), 7.88−7.94 (m, 4H), 7.76–7.85 (m, 3H), 7.59 (dd, *J*=3.76, 8.8 Hz, 2H), 4.93 (q, *J*=7.7 Hz, 1H), 3.92 (s, 3H), 3.78 (s, 3H), 3.19–3.38 (m, 2H). (^1^H,^13^C)‐HSQC NMR (400 MHz, [D_6_]DMSO) *δ*=129.1 (Ar), 128.9 (Ar), 128.7 (Ar), 126.1 (Ar), 126.0 (Ar), 125.9 (Ar), 119.8 (Ar), 118.9 (Ar), 115.1 (Ar), 110.4 (Ar), 60.8 (OMe), 60.5 (OMe), 54.7 (α‐C), 27.9 (β‐C); HRMS (ESI): *m/z* calcd for C_44_H_37_N_11_O_11_ [*M*+H]^+^ 896.2747; found 896.2734 (Δ*m*=−1.5 ppm), *t*_R_=7.34 min.

**Final derivative 13**: Synthetic protocol analogous to compound **3**. The title compound 15 was obtained as a colorless solid (18 mg, 14 % over two steps). ^1^H NMR (400 MHz, [D_6_]DMSO): *δ*=11.52 (s, 1H), 11.17 (s, 1H), 10.64 (s, 1H), 10.52 (s, 1H), 9.67 (s, 1H), 8.75 (d, *J*=7.3 Hz, 1H), 8.16–8.25 (m, 4H), 8.06 (d, *J*=9.0 Hz, 1H), 7.88–8.01 (m, 6H), 7.75–7.85 (m, 3H), 7.70 (s, 1H), 7.60 (d, *J*=2.5 Hz, 1H), 7.58 (d, *J*=2.5 Hz, 1H), 4.88–4.98 (m, *J*=6.0 Hz, 1H), 3.92 (s, 3H), 3.78 (s, 3H), 3.21–3.36 (m, 2H). (^1^H,^13^C)‐HSQC NMR (400 MHz, [D_6_]DMSO): *δ*=129.2 (Ar), 129.1 (Ar), 128.7 (Ar), 127.2 (Ar), 126.2 (Ar), 126.0 (Ar), 119.9 (Ar), 119.2 (Ar), 115.1 (Ar), 110.6 (Ar), 60.9 (OMe), 60.5 (OMe), 54.5 (α‐C), 27.7 (β‐C); HRMS (ESI): *m/z* calcd for C_43_H_36_N_12_O_11_ [*M*+H]^+^ 897.2699; found 897.2725 (Δ*m*=+2.9 ppm), *t*_R_=7.35 min.

**Final derivative 14**: Synthetic protocol analogous to compound **3**. The title compound 14 was obtained as a colorless solid (4 mg, 4 % over two steps). ^1^H NMR (400 MHz, [D_6_]DMSO) *δ*=10.55 (br s, 1H), 10.26 (s, 1H), 10.22 (s, 1H), 9.67 (br s, 1H), 9.29 (br s, 1H), 7.74–8.01 (m, 11H), 7.52–7.62 (m, 4H), 6.88 (br d, *J*=8.8 Hz, 2H), 4.77–4.87 (m, 1H), 3.86–3.92 (m, 3H), 3.76–3.81 (m, 3H). HRMS (ESI): *m/z* calcd for C_44_H_36_N_8_O_12_ [*M*+H]^+^ 869.2525; found 869.2521 (Δ*m*=−0.5 ppm), *t*_R_=7.82 min.

**Final derivative 15**: Synthetic protocol analogous to compound **3**. The title compound 15 was obtained as a colorless solid (15 mg, 25 % over two steps). ^1^H NMR (400 MHz, [D_6_]DMSO): *δ*=3.06−3.25 (m, 2H) 3.78 (s, 3H) 3.81 (s, 3H) 3.92 (s, 3H) 4.81 (q, *J*=7.9 Hz, 1H) 7.03 (d, *J*=8.8 Hz, 2H) 7.52−7.62 (m, 4H) 7.67 (br. s., 1H) 7.76 (d, *J*=8.8 Hz, 2H) 7.81 (d, *J*=8.8 Hz, 1H) 7.97 (d, *J*=8.8 Hz, 2H) 8.06 (d, *J*=9.0 Hz, 1H) 9.23 (d, *J*=8.0 Hz, 1H) 9.69 (s, 1H) 10.53 (s, 1H) 11.19 (s, 1H) 11.55 (s, 1H). ^13^C NMR (400 MHz, [D_6_]DMSO): *δ*=134.5 (Ar), 129.0 (Ar), 126.1 (Ar), 126.0 (Ar), 119.2 (Ar), 115.2 (Ar), 115.1 (Ar), 110.4 (Ar), 60.8 (OMe), 60.6 (OMe), 55.8 (OMe); HRMS (ESI): calcd for C_38_H_33_N_7_O_11_ [*M*+H]^+^ 764.2311; found 764.2316 (Δ*m*=+0.7 ppm).

## Conflict of interest

The authors declare no conflict of interest.

## Supporting information

As a service to our authors and readers, this journal provides supporting information supplied by the authors. Such materials are peer reviewed and may be re‐organized for online delivery, but are not copy‐edited or typeset. Technical support issues arising from supporting information (other than missing files) should be addressed to the authors.

Supplementary
