# Supplementary material for: Acetylenic Replacement of Albicidin's Methacrylamide Residue Circumvents Detrimental E/Z Photoisomerization and Preserves Antibacterial Activity
Source: Chemistry. 2021 May 21;27(35):9077–86. doi: 10.1002/chem.202100523 (PMC8362182; doi:10.1002/chem.202100523)
Supplement: Supplementary file 1 — Supplementary [file CHEM-27-9077-s001.pdf]

Chemistry–A European Journal

Supporting Information

Acetylenic Replacement of Albicidin's Methacrylamide Residue Circumvents Detrimental *E*/*Z* Photoisomerization and Preserves Antibacterial Activity

Iraj Behroz, Leonardo Kleebauer, Kay Hommernick, Maria Seidel, Stefan Grätz, Andi Mainz, John B. Weston, and Roderich D. Süssmuth*

Table of Contents

Procedures for Biological Assays. 2

NOE-Based Determination of the Isomeric State of Albicidin 4

Monitoring of (*E*)-(*Z*)-Isomerization for Fluoro-Albicidin by ^{1}H NMR Spectroscopy.... 5

MIC Values for the (*E*)- and (*Z*)-Isomers of Albicidin 6

Gyrase Inhibition Assay for the "Light" and "Dark" Samples of Albicidin.... 6

Synthetic Procedures 7

Proton and (^{1}H,^{13}C)-HSQC NMR Spectra of Albicidin Derivatives.... 20

- Compound 3.... 20
- Compound 4.... 21
- Compound 5.... 22
- Compound 6.... 23
- Compound 7.... 24
- Compound 8.... 25
- Compound 9.... 26
- Compound 10.... 27
- Compound 11.... 28
- Compound 12.... 29
- Compound 13.... 30
- Compound 14.... 31
- Compound 15.... 31

References 33

Author Contributions.... 33

Procedures for Biological Assays.

Microdilution Assay. Minimal inhibitory concentration (MIC) values were determined according to the ninth edition of the Approved Standard M07-A9. The test was carried out for six different bacterial strains (*E.coli* DSM1116 [Gram-negative], *E. coli* BW25113 [Gram-negative], *B. subtilis* DSM10 [Gram-postive], *M. luteus* DSM1790 [Gram-postive], *M. phlei* DSM750 [Gram-postive], *S. typhimurium* TA100 [Gram-negative]. 20 µL of cryo stock of each strain were inoculated in 20 mL LB medium (Lysogeny broth: 10 g/L peptone, 5 g/L yeast extract, 5 g/L NaCl) followed by incubation overnight at 37 °C, 200 rpm. The test inoculum was adjusted by the 0.5 McFarland Standard (OD_{625} from 0.08 to 0.1). Within 15 min of preparation, the adjusted inoculum suspension was diluted in MHBII (BBL™ Mueller-Hinton Broth II, Becton, Dickinson and Company, New Jersey, USA) so that each well contained approximately 5×10^5 CFU/mL in a final volume of 100 µL. 95 µL of the inoculum were applied per well and 5 µL of the (diluted) antibiotic substance were added. Previously, the dry antibiotic compounds (**1** to **15** and antibiotic control Ciprofloxacin (CIP)) were dissolved in DMSO (100%) with a concentration of 2560 µg/mL, and the so obtained stock solutions were further diluted in DMSO (100%). 5 µL of each antibiotic dilution were applied to the microdilution tray to reach final concentrations of 8 µg/mL to 0.016 µg/mL. One row of each well plate served as a growth control without antibiotic substances and another row of the microdilution tray served as sterility control (only MHB II-media). The antimicrobial effect of the solvent (DMSO) was tested by adding 5 µL DMSO to several wells. Purity check and cell titer control were performed according to International Standard M07-A9 on Mueller-Hinton II Agar (Mueller Hinton II Broth, 15 g/L agar-agar). Both microdilution trays and agar plates were incubated at 37 °C for 20 h and subsequently analyzed by naked eye. For determination of the MIC value of the (*Z*)-isomer, a freshly prepared sample of 2.56 mg/mL albicidin in DMSO-

d_6 was split into two aliquots ("Dark" vs. "Light"). A small volume of one aliquot ("Dark") was diluted in DMSO-d_6, protected from UV light and directly measured by ^{1}H NMR spectroscopy (100% (*E*)-isomer, no detectable amounts of (*Z*)). The rest of this aliquot was immediately stored in the dark. The other aliquot ("Light") was exposed to UV light (366 nm) for 15 h and then equally processed as the "Dark" sample. The 1D ^{1}H NMR spectrum revealed a relative composition of 20.7% (*E*)-isomer and 79.3% (*Z*)-isomer. Both stock solutions ("Dark" and "Light") were stored in UV-resistant vials in the dark until determination of MIC values. Given the apparent MIC value of the "Light" sample (MIC_{app}), the MIC value of (*E*)-albicidin ($MIC_{(E)}$) and the relative fractions of the two isomers ($f_{(E)}$ and $f_{(Z)}$) in the "Light" sample, we were able to calculate the actual MIC value of (*Z*)-albicidin ($MIC_{(Z)}$) according to the equation: $MIC_{(Z)} = (MIC_{app} - (f_{(E)}\ MIC_{(E)})) / f_{(Z)}$.

DNA Gyrase Inhibition Assay. DNA-supercoiling experiments with DNA-gyrase were performed in a total volume of 30 µL gyrase buffer (protocol by Inspiralis Limited, Norwich, UK). The incubations contained 0.5 µg relaxed pBR322 plasmid DNA (inspiralis Limited), 1 U DNA-gyrase (6 U/µL) (Inspiralis Limited) and the albicidins **1−15** at a final concentration of 45 nM. The final DMSO concentration was 3%. Samples were incubated at 37 °C for 30 min and subsequently loaded on an agarose gel. Electrophoretic analysis was performed using a 1% agarose gel (100 V, 90 min). DNA bands were stained with ethidium bromide and visually analyzed.

NOE-Based Determination of the Isomeric State of Albicidin

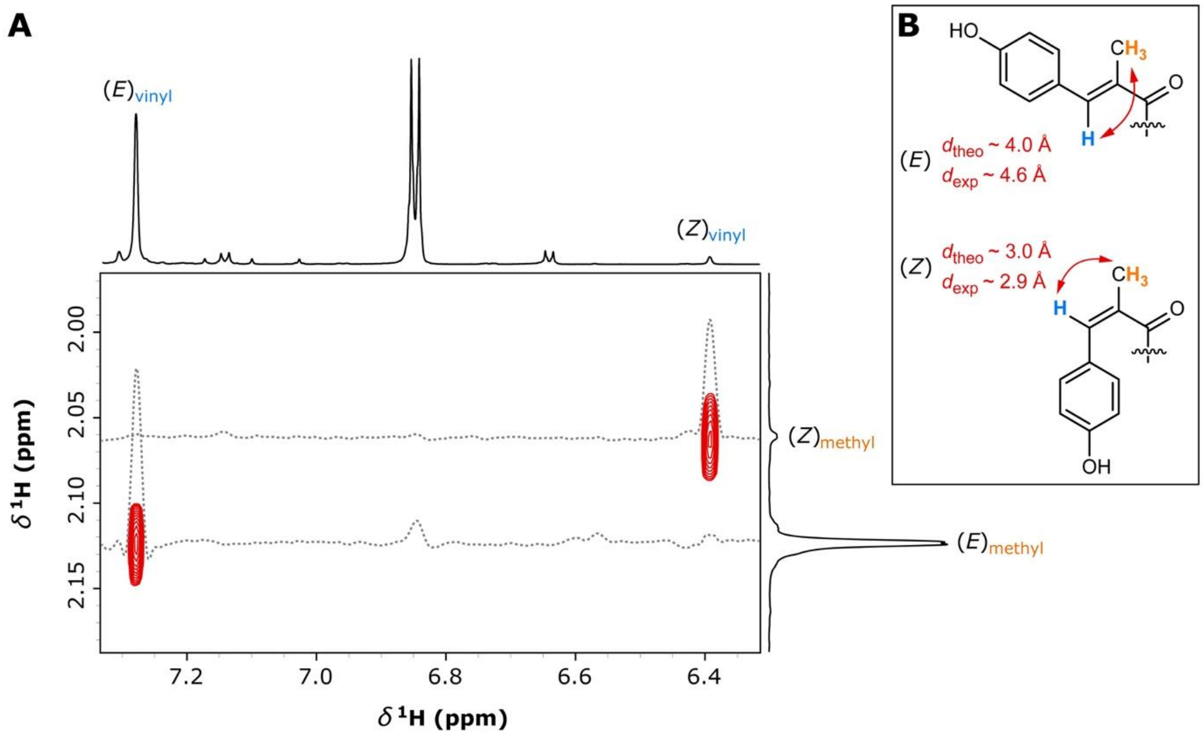

Figure S1. NOE-based determination of the isomeric state of albicidin. **A)** Section of a ^1H-^1H ROESY spectrum recorded on a sample of albicidin in DMSO-d_6 after short UV exposure. The relative (*E*)-to-(*Z*) ratio amounted to approx. 18 based on signal integration of the corresponding 1D ^1H spectrum (relevant regions shown on *top* and *right*). Relevant signals for the two isomers are labeled. Additionally, 1D projections for the two methyl signals are depicted as dashed lines to better visualize the NOE cross-peak intensities. **B)** Chemical structures of the two isomers of MCA and relative positions of vinyl and methyl protons (same color code as in A). Theoretical (d_{theo}) and experimentally determined distances (d_{exp}) through semiquantitative NOE analysis are given for the (*E*)- and (*Z*)-isomer, respectively.

Monitoring of (*E*)-(*Z*)-Isomerization for Fluoro-Albicidin by ^{1}H NMR Spectroscopy

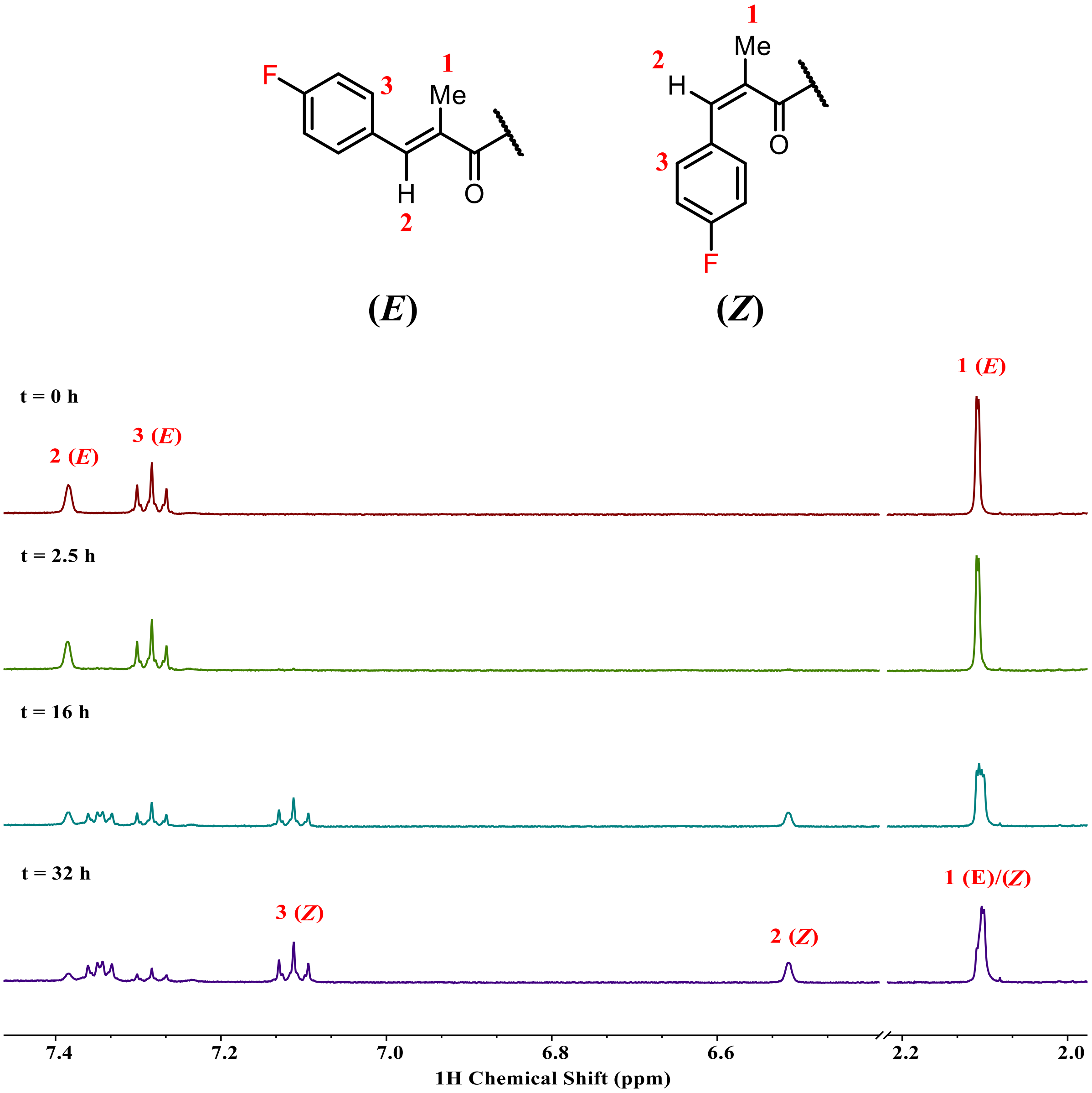

Figure S2. Photochemical (*E*)-(*Z*)-isomerization of fluoro-albicidin's cinnamoyl residue (building block A) monitored by ^{1}H NMR spectroscopy in a time span of 32 h. Shown are the spectral regions of the aromatic/vinylic (*left*) and methyl protons (*right*). The two resonance sets for the (*E*)- and (*Z*)-isomers are labeled accordingly.

MIC Values for the (*E*)- and (*Z*)-Isomers of Albicidin

Table S1. MIC (µg · mL^{-1}) values for ciprofloxacin (CIP), albicidin (**1**), albicidin "Light", and albicidin "Dark" samples.[a]

strain	CIP	albicidin	albicidin "Light" (*E*)-isomer (20.7%) (*Z*)-isomer (79.3%)	albicidin "Light" mean value	albicidin "Dark" (*E*)-isomer (100%)	(Z)-isomer (calc.)
E. coli **BW25113**	≤ 0.016<br>≤ 0.016	0.063<br>0.063	0.25<br>0.25	0.25	0.063<br>0.063	0.2988
E. coli **DSM1116**	0.016<br>0.031	0.063<br>0.063	0.25<br>0.5	0.375	0.063<br>0.063	0.4564
S. typhimurium **TA100**	≤ 0.016<br>≤ 0.016	≤ 0.016<br>≤ 0.016	0.063<br>0.125	0.094	0.016<br>0.016	0.1144
B. subtilis **DSM10**	0.25<br>0.25	0.25<br>0.25	1.0<br>1.0	1.0	0.25<br>0.25	1.1958
M. luteus **DSM1790**	1.0<br>1.0	1.0<br>1.0	8.0<br>8.0	8.0	1.0<br>1.0	9.8272

Gyrase Inhibition Assay for the "Light" and "Dark" Samples of Albicidin

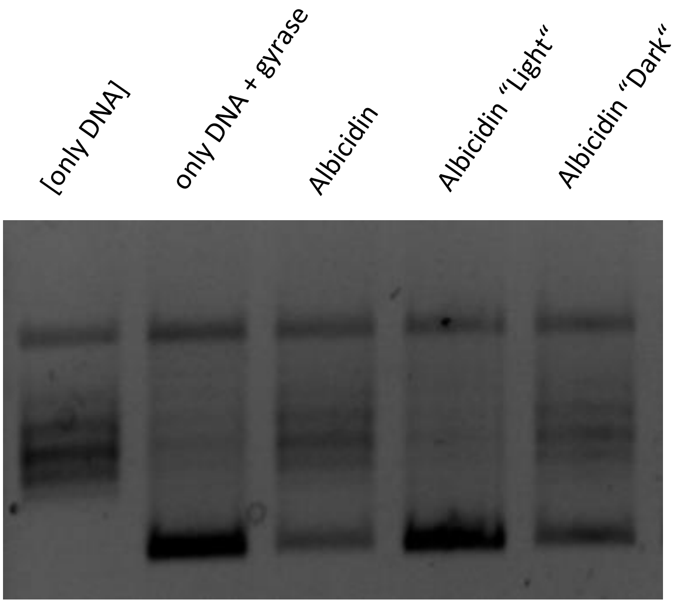

Figure S3. DNA gyrase inhibition assay for albicidin (1), albicidin "Light" and albicidin "Dark" samples. The control experiment without enzyme and drug (left lane) shows relaxed DNA. Addition of DNA gyrase results in supercoiled DNA (second lane from left). All samples were tested at a concentration of 45 nM.

Synthetic Procedures

General Information. Commercially available reagents (*Carl Roth GmbH* and *Co. KG*, Karlsruhe, Germany; *Sigma-Aldrich*, Taufkirchen, Germany; *Iris Biotech GmbH*, Marktredwitz, Germany; *Orpegen*, Heidelberg, Germany; *ABCR*, Karlsruhe, Germany; *Alfa Aesar*, Karlsruhe, Germany; *Merk*, Darmstadt, Germany; *TCI*, Eschborn, Germany; *VWR International GmBH*, Darmstadt, Germany; *Acros*, Geel, Belgium) and solvents (*Fisher Scientific-Acros*, Schwerte, Germany) were used without further purification. Whenever necessary, reactions were carried out under an atmosphere of argon or nitrogen and in dry solvents. HPLC solvents (*Fisher Scientific-Acros*, Schwerte, Germany) and NMR solvents (*Deutero GmbH*, Kastellaun, Germany; *Sigma-Aldrich*, Taufkirchen, Germany) were used without further purification. Reactions and purifications were monitored by analytical thin layer chromatography (TLC) on aluminium-backed plates coated with *Macherey-Nagel* silica gel (60, F254) using solvent systems based on ethyl acetate, *n*-hexane, dichloromethane, and methanol. Analysis was performed by visualizing under UV light (λ = 254 nm), by staining with $KMnO_4$-solution ($KMnO_4$ (3 g), K_2CO_3 (20 g, H_2O (300 mL), 5% $NaOH_{(aq.)}$ (5 mL)) and with ninhydrin-solution (ninhydrin (0.3 g), AcOH (3 mL), *n*-BuOH (100 mL)). Flash chromatography was performed on silica gel (particle size 40−63 μm, *VWR Chemicals*, Darmstadt, Germany) and solvent mixtures based on ethyl acetate, *n*-hexane, dichloromethane, and methanol. Preparative HPLC was carried out on a 1260 Infinity (*Agilent Technologies*, Waldbronn, Germany) system with a polymeric reversed phase column (PLRP-S 100A, 300 x 50 mm, particle size 10 μm, *Agilent Technologies*, Waldbronn, Germany). Eluent A was water with 0.1% trifluoroacetic acid and eluent B was acetonitrile with 0.1% trifluoroacetic acid. A flow rate of 70 mL min^{-1} was used. ^{1}H and ^{13}C NMR spectra were recorded at 298 K using Bruker Avance-II 400 MHz, Bruker Avance-III 500 MHz or Bruker Avance-III 700 MHz instruments

(*Bruker*, Karlsruhe, Germany). The chemical shifts are reported in parts per million (ppm) using the residual solvent peak as an internal reference (DMSO-d_6, C$D$$Cl_3$). Multiplicity (br. s = broad singlet, s = singlet, d = doublet, dd = doublet of doublet, t = triplet, q = quartet, m = multiplet) and coupling constants (J = Hz) are quoted where possible. NMR spectra were analyzed using TopSpin3.1 (*Bruker Biospin, Karlsruhe, Germany*), the ACD/Spectrus Processor (*ACD/Labs*, Toronto, Ontario, Canada) or MestReNova (*Mestrelab Research S.L.*, Santiago de Compostela, Spain). 2D NOESY and ROESY spectra were recorded with mixing times of 400 ms and 300 ms, respectively. Cross-peak volumes V were normalized to the corresponding diagonal peak volumes. Distances d_{exp} were approximated using the equation $d_{exp} = d_{ref} (V_{ref} / V_{exp})^{1/6}$, where d_{ref} and V_{ref} represent the reference distance (2.48 Å) and peak volume, respectively, of the correlation peaks between the two ortho-protons in MCA. HPLC-ESI-HRMS spectra were recorded on a QTrap LTQ XL (*Thermo Fisher Scientific*, Waltham, Massachusetts, USA) with an Agilent 1200 Series HPLC-System (*Agilent Technologies*, Waldbronn, Germany) using a reversed-phase C18 column (Hypersil 100, 150 x 4.6 mm, particle size 5 µm, *Thermo Fisher Scientific*, Waltham, Massachusetts, USA). Eluent A was water with 0.1% formic acid; eluent B was methanol with 0.1% formic acid. A flow rate of 3 mL min^{-1} was used. HRMS spectra were analyzed using Xcalibur (*Thermo Fisher Scientific GmbH*, Bremen, Germany). All biologically tested compounds had a purity of >95%. UV irradiation experiments to investitage the photochemical (*E*)-(*Z*)-isomerization of albicidin's cinnamoyl residue were conducted using a F8T5/BLB fluorescence lamp (nominal wattage: 8 W; target irradiation: 366 nm) (OSRAM Sylvania Inc.).

Methyl 4-((4-acetoxyphenyl)ethynyl)benzoate (18)

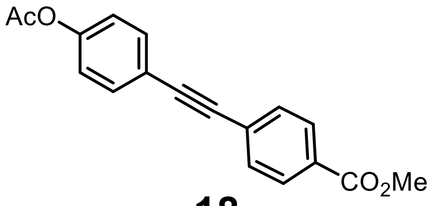

18

To a solution of methyl 4-iodobenzoate (**17**) (245 mg, 1.53 mmol, 1.00 equiv) in Et_3N (15 mL) were added $Pd(PPh_3)_2Cl_2$ (10.7 mg, 15.3 µmol, 0.01 equiv) and CuI (2.91 mg, 15.3 µg, 0.01 equiv) at room temperature. A solution of 4-ethynylphenyl acetate (**16**) (481 mg, 1.84 mmol, 1.20 equiv) in Et_3N (3 mL) was added dropwise to the first solution and the reaction mixture was stirred at room temperature for 16 h. The solution was diluted with toluene (30 mL) and washed with 10% $HCl_{(aq.)}$ (3×) followed by brine. The organic phase was dried over anhyd. Na_2SO_4 and concentrated under reduced pressure. Purification of the crude product by column chromatography on silica gel (*n*-hexane/EtOAc, 7:1–5:1) afforded the desired product **18** (308 mg, 1.05 mmol, 69%) as an off-white solid. ^1H NMR (500 MHz, C*D*Cl_3): δ=8.05–7.97 (m, 2H), 7.61–7.51 (m, 4H), 7.14–7.07 (m, 2H), 3.93 (s, 3H), 2.31 (s, 3H). ^{13}C NMR (126 MHz, C*D*Cl_3): δ=169.2, 166.7, 151.0, 133.1, 131.6, 129.7, 129.7, 128.0, 122.0, 120.5, 91.6, 88.8, 52.4, 21.3. HRMS (ESI): *m*/*z* calcd for $C_{18}H_{14}O_4$ $[M+H]^+$ 295.0965; found 295.0962 (Δm = −1.0 ppm), t_R = 10.4 min.

4-((4-Methoxyphenyl)ethynyl)benzoic acid (22)

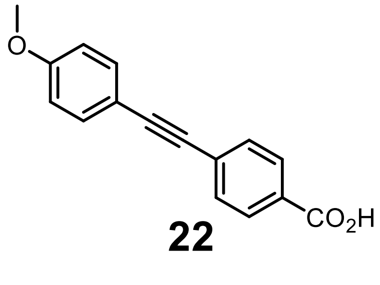

22

1-Ethynyl-4-methoxybenzene (**21**) (1.00 g, 7.56 mmol, 1.10 equiv) and methyl 4-iodobenzoate (**17**) (1.80 g, 6.87 mmol, 1.00 equiv) were dissolved in dry THF (10 mL) and $Pd(PPh_3)Cl_2$ (0.241 g, 0.344 mmol, 0.050 equiv), CuI (0.078 g, 0.412 mmol, 0.060 equiv), and pyridine (1.5 mL) were successively added at 0 °C. After stirring for 2 h at room temperature, the reaction mixture was diluted with toluene (30 mL), washed with 1 N $HCl_{(aq.)}$ and H_2O (2×), dried over anhyd. $MgSO_4$, and concentrated *in vacuo.* The crude product was purified by trituration with MeOH and subsequent filtration through a pad of celite to afford methyl

4-((4-methoxyphenyl)ethynyl)benzoate (1.45 g, 5.43 mmol, 79%) as a brown solid. 4-((4-methoxyphenyl)ethynyl)benzoate (1.40 g, 5.26 mmol, 1.00 equiv) was then dissolved in a mixture of THF (15 mL) and MeOH (15 mL) and 3 N $KOH_{(aq.)}$ (15 mL) was added dropwise. The reaction mixture was stirred at room temperature for 3 h before the organic solvents were removed *in vacuo*, the aq. residue was diluted with H_2O and acidified to pH ≈ 2 by the addition of 10% $HCl_{(aq)}$. The solution was extracted with EtOAc (3×) and the combined organic phases were dried over anhyd. $MgSO_4$ and concentrated under reduced pressure to obtain the analytically pure title compound **22** (1.32 g, 5.26 mmol, quant.) as a brown solid. ^{1}H NMR (400 MHz, [D$_6$]DMSO): δ=13.12 (s, 1H), 7.99–7.91 (m, 2H), 7.67–7.59 (m, 2H), 7.57–7.49 (m, 2H), 7.05–6.96 (m, 2H), 3.80 (s, 3H). ^{13}C NMR (101 MHz, [D$_6$]DMSO): δ=166.7, 159.9, 133.2, 131.3, 130.2, 129.6, 127.1, 114.5, 113.7, 92.4, 87.4, 55.3, 40.2, 40.2, 39.9, 39.7, 39.5, 39.3, 39.1, 38.9. HRMS (ESI): *m*/*z* calcd for $C_{16}H_{12}O_3$ [*M*+H]$^+$ 253.0859; found 253.0859 (Δm = ±0.0 ppm), t_R = 9.14 min.

Methyl 4-((6-fluoropyridin-3-yl)ethynyl)benzoate (25)

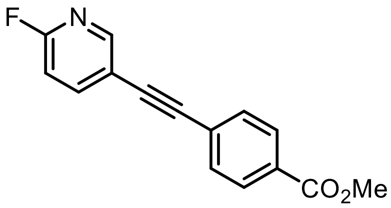

25

To a solution of 2-fluoro-5-iodopyridine (**23**) (500 mg, 2.24 mmol, 1.00 equiv) in dry THF (10 mL) were successively added Et_3N (5 mL), $Pd(PPh_3)_2Cl_2$ (15.7 mg, 22.4 µmol, 0.01 equiv), CuI (4.27 mg, 22.4 µg, 0.01 equiv) and methyl 4-ethynylbenzoate (**24**) (431 mg, 2.69 mmol, 1.20 equiv). The reaction mixture was stirred at room temperature for 16 h, then diluted with toluene (30 mL) and washed with 10% $HCl_{(aq.)}$ (3×) followed by brine. The organic phase was dried over anhyd. Na_2SO_4 and concentrated under reduced pressure. Purification of the crude product by column chromatography on silica gel (*n*-hexane/EtOAc, 12:1–8:1) afforded the desired product **25** (561 mg, 2.20 mmol, 98%) as a yellow solid. ^{1}H NMR (500 MHz, C*D*Cl$_3$): δ=8.41–8.39 (m, 1H), 8.05–8.02 (m, 2H),

7.93–7.88 (m, 1H), 7.60–7.56 (m, 2H), 6.97–6.93 (m, 1H), 3.92 (d, *J* = 3.0 Hz, 3H). HRMS (ESI): *m*/*z* calcd for $C_{15}H_{10}FNO_2$ [*M*+H]$^+$ 256.0768, found 256.0767 (Δm = −0.4 ppm), t_R = 9.75 min.

4-((6-Methoxypyridin-3-yl)ethynyl)benzoic acid (26)

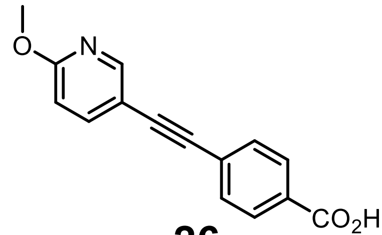

26

To a solution of fluoropyridine **25** (500 mg, 1.96 mmol, 1.00 equiv) in a mixture of MeOH (25 mL) and THF (25 mL) was added 3 N $KOH_{(aq.)}$ (25 mL) and the reaction mixture was stirred at room temperature for 16 h. The solution was then acidified to pH ≈ 2 by the addition of 1 N $HCl_{(aq.)}$ and the organic solvent evaporated under reduced pressure. The aq. residue was diluted with H_2O and extracted with $CHCl_3$ (3×). The combined organic phases were dried over anhyd. Na_2SO_4 and concentrated *in vacuo* to give the pure benzoic acid **26** (470 mg, 1.96 mmol, quant.) as a colourless solid. ^{1}H NMR (400 MHz, [D$_6$]DMSO): δ=12.29 (br. s, 1H), 8.46–8.40 (m, 1H), 7.98 (d, *J* = 8.0 Hz, 2H), 7.90 (dd, *J* = 8.6, 2.4 Hz, 1H), 7.63 (d, *J* = 8.0 Hz, 2H), 6.90 (dd, *J* = 8.6, 0.8 Hz, 1H), 3.89 (s, 3H). ^{13}C NMR (101 MHz, [D$_6$]DMSO) δ=163.2, 150.1, 141.6, 131.2, 129.6, 112.0, 110.9, 90.1, 88.8, 53.6, 21.1. HRMS (ESI): *m*/*z* calcd for $C_{15}H_{11}NO_3$ [*M*−H]$^-$ 252.0666; found 252.0669 (Δm = +1.2 ppm), t_R = 8.14 min.

Methyl 4-((4-fluorophenyl)ethynyl)benzoate (28)

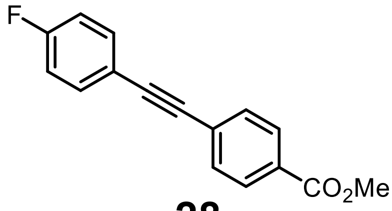

28

To a solution of 1-fluoro-4-iodobenzene (**27**) (500 mg, 2.25 mmol, 1.00 equiv) in dry THF (10 mL) and Et_3N (5 mL), $Pd(PPh_3)_2Cl_2$ (15.8 mg, 22.5 µmol, 0.01 equiv), CuI (4.29 mg, 22.5 µmol, 0.01 equiv), and methyl 4-ethynylbenzoate (**24**) (433 mg, 2.70 mmol, 1.20 equiv). The reaction mixture was stirred at room temperature for 16 h, then diluted with toluene (30 mL) and washed

with 10% $HCl_{(aq.)}$ (3×) followed by brine. The organic phase was dried over anhyd. Na_2SO_4 and concentrated under reduced pressure. The crude product was purified by column chromatography on silica gel (*n*-hexane/EtOAc, 12:1–8:1) to obtain the title compound **28** (289 mg, 1.13 mmol, 50%) as a colourless solid. ^{1}H NMR (500 MHz, C*D*Cl_3): δ=8.04–7.99 (m, 2H), 7.59–7.54 (m, 2H), 7.55–7.50 (m, 2H), 7.09–7.03 (m, 2H), 3.93 (s, 3H). ^{13}C NMR (126 MHz, C*D*Cl_3): δ=166.7, 163.9, 161.9, 133.8, 133.8, 131.6, 129.7, 129.7, 128.0, 119.0, 119.0, 116.0, 115.8, 91.4, 88.5, 52.4. HRMS (ESI): *m*/*z* calcd for $C_{16}H_{11}FO_2$ $[M+H]^+$ 255.0816; found 255.0815 (Δm = −0.4 ppm), t_R = 11.0 min.

4-((4-Fluorophenyl)ethynyl)benzoic acid (29)

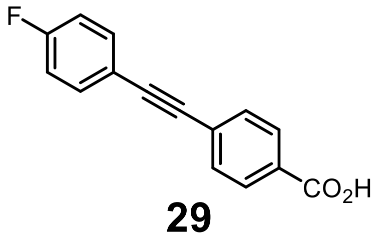

29

The diaryl alkyne **28** (250 mg, 980 µmol, 1.00 equiv) was dissolved in a mixture of THF (30 mL) and MeOH (30 mL) and 3 N $KOH_{(aq.)}$ (15 mL) was added dropwise at room temperature. After stirring the reaction mixture for 16 h at room temperature, the solution was acidified to pH ≈ 2 by the addition of 1 N $HCl_{(aq.)}$ before evaporating the organic solvents under reduced pressure. The aq. residue was diluted with H_2O and extracted with EtOAc (3×). The combined organic phases were dried over anhyd. Na_2SO_4 and concentrated under reduced pressure. Drying of the crude product under high vacuum yielded the analytically pure title compound **29** (224 mg, 934 µmol, 95%) as a colourless solid. ^{1}H NMR (400 MHz, $[D_6]$DMSO): δ=13.11 (s, 1H), 8.01–7.93 (m, 2H), 7.70–7.61 (m, 4H), 7.35–7.25 (m, 2H). ^{13}C NMR (101 MHz, $[D_6]$DMSO): δ=166.7, 163.6, 161.1, 134.0, 134.0, 131.5, 130.6, 129.6, 126.5, 118.3, 118.3, 116.3, 116.1, 91.0, 88.4. HRMS (ESI): *m*/*z* calcd for $C_{15}H_9FO_2$ $[M-H]^-$ 239.0514; found 239.0512 (Δm = −0.8 ppm), t_R = 8.80 min.

4-((4-Isocyanophenyl)ethynyl)benzoic acid (32)

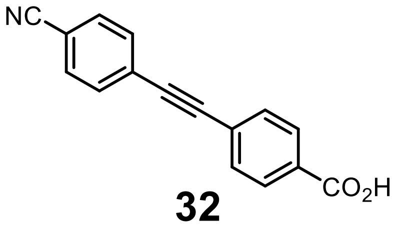

32

To a solution of 1-iodo-4-isocyanobenzene (**30**) (1.20 g, 5.24 mmol, 1.00 equiv) in Et_3N (10 mL) were added $Pd(PPh_3)_4$ (60.5 mg, 52.4 µmol, 0.01 equiv), CuI (9.98 mg, 52.4 µmol, 0.01 equiv), and 4-ethynylbenzoic acid (**31**) (1.01 mg, 6.29 mmol, 1.20 equiv). The reaction mixture was stirred at room temperature for 16 h, then diluted with toluene (30 mL) and washed with 10% $HCl_{(aq.)}$ (3×) followed by brine. The organic phase was dried over anhyd. Na_2SO_4 and concentrated under reduced pressure. The crude product was purified by column chromatography on silica gel (*n*-hexane/EtOAc, 6:1–3:1) to obtain the title compound **32** (1.52 g, 4.77 mmol, 91%) as a beige-coloured solid. ^{1}H NMR (500 MHz, C*D*Cl_3): δ=8.07–8.02 (m, 2H), 7.68–7.63 (m, 2H), 7.63–7.56 (m, 4H), 3.93 (s, 3H). ^{13}C NMR (126 MHz, C*D*Cl_3): δ=166.5, 132.3, 132.2, 131.8, 130.4, 129.8, 127.7, 126.9, 118.5, 112.2, 92.8, 90.4, 52.4. HRMS (ESI): *m*/*z* calcd for $C_{17}H_{11}NO_2$ $[M+H]^+$ 262.0863; found 262.0861 (Δm = −0.8 ppm).

Perchlorophenyl 4-((4-isocyanophenyl)ethynyl)benzoate (33)

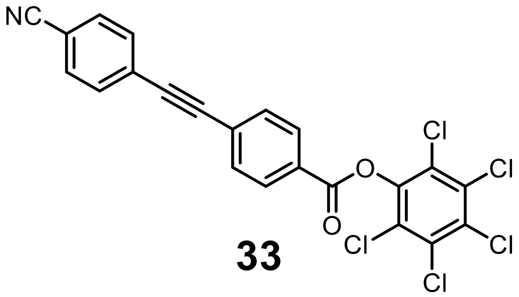

33

The diaryl alkyne **32** (990 mg, The diaryl alkyne **32** (200 mg, 0.809 mmol, 1.00 equiv), pentachlorophenol (259 mg, 0.971 mmol, 1.20 equiv) DCC (217 mg, 1.05 mmol, 1.30 equiv) and DMAP (9.88 mg, 80.9 µmol, 0.100 equiv) were dissolved in DMF (8 mL) and the reaction mixture was stirred at room temperature for 16 h. After removing DMF *in vacuo*, the residue was taken up CH_2Cl_2 and washed with 10% $HCl_{(aq.)}$ (3×) followed by brine. The organic phase was dried over anhyd. Na_2SO_4 and concentrated under reduced pressure to obtain the crude product, which was purified by column chromatography on silica gel (*n*-

hexane/EtOAc, 4:1–2:1) to afford the title compound **33** (150 mg, 307 mmol, 65% over two steps) as a colourless solid. ^{1}H NMR (400 MHz, C*D*Cl$_3$): δ=8.27–8.20 (m, 2H), 7.75–7.62 (m, 6H). ^{13}C NMR (101 MHz, C*D*Cl$_3$): δ=162.0, 144.2, 132.4, 132.2, 132.2, 132.1, 131.8, 130.7, 128.7, 127.9, 127.4, 127.3, 118.4, 112.4, 92.2, 91.5.

4-(4-Hydroxyphenethyl)benzoic acid (37)

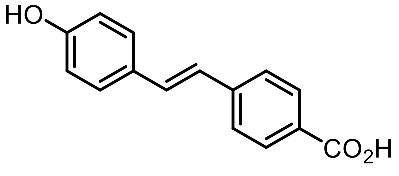

37

The diaryl alkyne **36** (350 mg, 1.46 mmol, 1.00 equiv) was dissolved in dry THF (5 mL) and the solution was purged with argon for 10 min. Palladium (10wt.% on activated carbon, 155 mg) was added, the reaction mixture was saturated with H_2 and stirred at room temperature for 5 h under hydrogen atmosphere. The suspension was filtered over a pad of Celite®, concentrated under reduced pressure, and the residue was dried under high vacuum to give the title compound **37** (317 mg, 1.31 mmol, 90%) as a colourless powder. ^{1}H NMR (500 MHz, [D$_6$]DMSO): δ=12.75 (br s, 1H), 9.12 (s, 1H), 7.82–7.86 (m, 2H), 7.32 (d, J = 8.4 Hz, 2H), 6.97–7.02 (m, 2H), 6.64–6.66 (m, 2H), 2.76–2.93 (m, 4H). ^{13}C NMR (126 MHz, [D$_6$]DMSO): δ=167.8 (CO_2H), 155.8 (COH), 147.6 (Ar), 131.7 (Ar), 129.7 (C*H*-Ar), 129.7 (C*H*-Ar), 129.1 (C*H*-Ar), 115.5 (C*H*-Ar), 37.8 (CH_2), 36.3 (CH_2). HRMS (ESI): m/z calcd for $C_{15}H_{13}O_3$ $[M{-}H]^-$ 241.0870; found 241.0864 (Δm = −2.4 ppm).

Methyl 4-(4-ethynylbenzamido)benzoate (45)

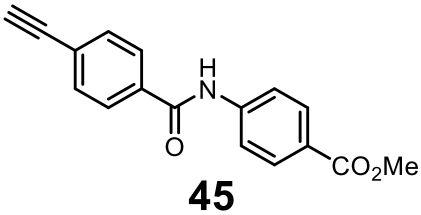

45

BTC (870 mg, 2.93 mmol, 0.45 equiv) was added to a solution of 4-ethynylbenzoic acid (1.43 g, 9.77 mmol, 1.50 equiv) in dry THF (25 mL) and the solution was cooled down to 0 °C. 2,4,6-collidine (10.4 mL, 78.2 mmol, 12.0 equiv) was added dropwise and the resulting suspension was stirred at that temperature for 45 min. Subsequently, a premixed solution of methyl

4-aminobenzoate (985 mg, 6.52 mmol, 1.00 equiv) and DIPEA (11.4 mL, 65.2 mmol, 10.0 equiv) in dry THF (15 mL) was added dropwise to the suspension and the reaction mixture was stirred for 16 h while warming up to room temperature. After removing all volatiles under reduced pressure, the residue was taken up in EtOAc and washed with 1 N $HCl_{(aq.)}$ (3×), satd. aq. $NaHCO_3$ (3×), and brine. The organic phase was dried over anhyd. Na_2SO_4 and concentrated *in vacuo* to obtain the crude product, which was purified by column chromatography on silica gel (0.3–1% MeOH in CH_2Cl_2) to afford the desired product **45** (0.859 g, 3.06 mmol, 47%) as colourless solid. ^{1}H NMR (400 MHz, [D$_6$]DMSO): δ=10.64 (s, 1H), 8.02–7.92 (m, 6H), 7.68–7.62 (m, 2H), 4.44 (s, 1H), 3.83 (s, 3H). ^{13}C NMR (101 MHz, [D$_6$]DMSO): δ=165.8, 165.2, 143.5, 134.6, 131.8, 130.2, 128.2, 125.1, 124.5, 119.7, 83.3, 82.8, 52.0, 40.2, 40.2, 39.9, 39.7, 39.5, 39.3, 39.1, 38.9, 7.4. HRMS (ESI): *m*/*z* calcd for $C_{17}H_{13}NO_3$ [*M*+H]$^+$ 280.0968; found 280.0967 (Δm = −0.4 ppm).

Perchlorophenyl 4-(4-ethynylbenzamido)benzoate (46)

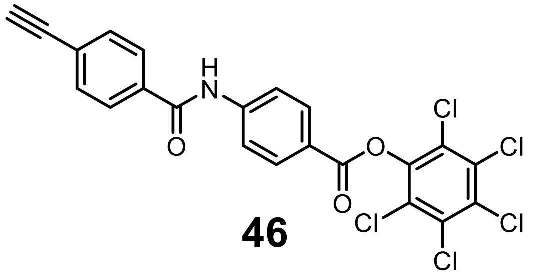

46

To a solution of methyl 4-(4-ethynylbenzamido)benzoate (**45**) (830 mg, 2.97 mmol, 1.00 equiv) in a mixture of THF (20 mL) and MeOH (20 mL) was added 3 N $KOH_{(aq.)}$ (15 mL) and the reaction mixture was stirred at room temperature for 3 h. After removing the organic solvents *in vacuo*, the residue was taken up in EtOAc and washed with 1 N $HCl_{(aq.)}$ (3×) and brine. The organic phase was dried over anhyd. Na_2SO_4 and concentrated under reduced pressure to obtain the crude product, which was purified by column chromatography on silica gel (3–20% MeOH in CH_2Cl_2) to afford 4-(4-ethynylbenzamido)benzoic acid (680 mg, 2.55 mmol, 86%). ^{1}H NMR (400 MHz, [D$_6$]DMSO): δ=12.39 (br. s, 1H), 10.41 (s, 1H), 8.01–7.81 (m, 6H), 7.55–7.51 (m, 2H), 3.85 (d, *J* = 1.1 Hz, 1H). ^{13}C NMR (101 MHz, [D$_6$]DMSO): δ=166.9, 164.9, 142.8,

134.4, 131.3, 129.9, 127.7, 125.5, 125.0, 119.2, 82.3, 81.5. HRMS (ESI): *m*/*z* calcd for $C_{16}H_{11}NO_3$ [*M*+H]$^+$ 266.0812; found 266.0811 (Δm = −0.4 ppm). To a solution of 4-(4-ethynylbenzamido)benzoic acid (150 mg, 566 µmol, 1.00 equiv) in DMF (5 mL) were successively added pentachlorophenol (166 mg, 622 µmol, 1.10 equiv), DCC (140 mg, 679 µmol, 1.20 equiv), and DMAP (6.91 mg, 56.6 µmol, 0.10 equiv) and the reaction mixture was stirred at room temperature for 16 h. After removing DMF under high vacuum, the residue was taken up in EtOAc and washed with 10% $HCl_{(aq.)}$ (3×) followed by brine. The organic phase was dried over anhyd. Na_2SO_4 and concentrated under reduced pressure to obtain the crude product, which was purified by column chromatography on silica gel (0.3–15% MeOH in CH_2Cl_2) to afford the title compound **46** (171 mg, 334 mmol, 59%) as a colourless solid. ^{1}H NMR (400 MHz, [D$_6$]DMSO): δ=10.81 (s, 1H), 8.25–8.17 (m, 2H), 8.13–8.05 (m, 2H), 8.05–7.97 (m, 2H), 7.70–7.63 (m, 2H), 4.46 (s, 1H). ^{13}C NMR (101 MHz, [D$_6$]DMSO): δ=165.3, 161.8, 150.4, 145.4, 144.1, 134.3, 131.8, 131.6, 131.3, 130.34, 128.3, 127.5, 125.3, 121.4, 120.8, 120.0, 83.5, 82.8.

Methyl 4-(4-cyanobenzamido)benzoate (48)

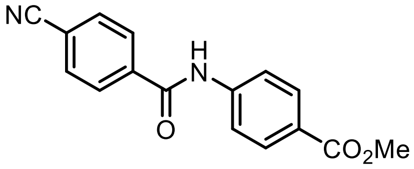

48

BTC (442 mg, 1.49 mmol, 0.45 equiv) was added to a solution of 4-cyanobenzoic acid (**47**) (730 mg, 4.96 mmol, 1.50 equiv) in dry THF (7 mL) and the solution was cooled down to 0 °C. 2,4,6-collidine (5.26 mL, 39.7 mmol, 12.0 equiv) was added dropwise and the resulting suspension was stirred at that temperature for 45 min. Subsequently, a premixed solution of methyl 4-aminobenzoate (500 mg, 3.31 mmol, 1.00 equiv) and DIPEA (5.76 mL, 33.1 mmol, 10.0 equiv) in dry THF (4 mL) was added dropwise to the suspension and the reaction mixture was stirred for 16 h while warming up to room temperature. After removing all volatiles under reduced pressure, the residue was taken up in EtOAc and washed with

1 N $HCl_{(aq.)}$ (3×), satd. aq. $NaHCO_3$ (3×), and brine. The organic phase was dried over anhyd. Na_2SO_4 and concentrated *in vacuo* to obtain the crude product, which was purified by recrystallization (*n*-hexane/EtOAc, 5:1) to afford the title compound **48** (578 mg, 2.02 mmol, 61%) as colourless solid. ^{1}H NMR (400 MHz, [D$_6$]DMSO): δ=10.78 (s, 1H), 8.15–8.07 (m, 2H), 8.07–8.01 (m, 2H), 8.01–7.91 (m, 4H), 3.84 (s, 3H). ^{13}C NMR (101 MHz, [D$_6$]DMSO): δ=165.8, 164.6, 143.2, 138.5, 132.5, 130.1, 128.6, 124.7, 119.7, 118.2, 114.1, 51.9.

Methyl 4-(4-(1*H*-tetrazol-5-yl)benzamido)benzoate (49)

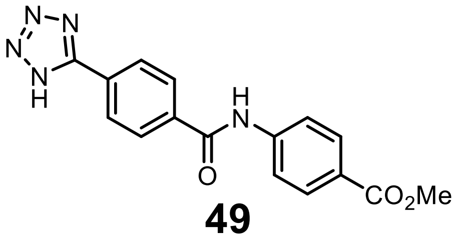

49

Methyl 4-(4-cyanobenzamido)benzoate (**48**) (200 mg, 714 µmol, 1.00 equiv), NaN_3 (58.0 mg, 892 µmol, 1.25 equiv), and L-proline (24.7 mg, 214 µmol, 0.30 equiv) were dissolved in DMF (6 mL) and stirred at 150 °C for 16 h. The solution was poured into ice-water (15 mL) under vigorous stirring and subsequently acidified to pH ≈ 2 by the addition of conc. HCl(aq.). The precipitate was isolated by filtration through a sintered funnel and dried under high vacuum to afford the pure title compound **49** (120 mg, 371 µmol, 52%) as a colourless solid. ^{1}H NMR (400 MHz, [D$_6$]DMSO): δ=10.73 (s, 1H), 8.26–8.14 (m, 4H), 8.02–7.93 (m, 4H), 3.84 (s, 3H). ^{13}C NMR (101 MHz, [D$_6$]DMSO): δ=165.8, 165.2, 143.5, 136.7, 130.2, 128.9, 127.3, 127.0, 124.5, 119.7, 52.0. HRMS (ESI): *m*/*z* calcd for $C_{16}H_{13}N_5O_3$ $[M+H]^+$ 324.1091; found 324.1090 (Δm = +0.3 ppm).

4-(4-(1*H*-Tetrazol-5-yl)benzamido)benzoic acid (50)

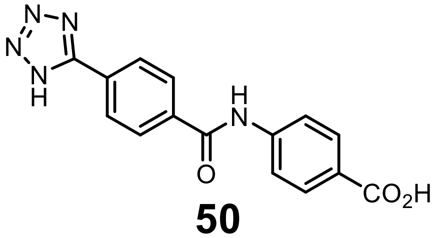

50

To a solution of methyl 4-(4-(1*H*-tetrazol-5-yl)benzamido)benzoate (**49**) (120 mg, 371 µmol, 1.00 equiv) in a mixture of THF (3 mL) and MeOH (3 mL) was added 3 N $KOH_{(aq.)}$ (3 mL) and the

reaction mixture was stirred at room temperature for 1 h. After removing the organic solvents *in vacuo*, the residue was taken up in EtOAc and washed with 1 N $HCl_{(aq.)}$ (3×) and brine. The organic phase was dried over anhyd. Na_2SO_4 and concentrated under reduced pressure to obtain the title compound **50** (92.0 mg, 297 µmol, 80%) as a colourless solid. HRMS (ESI): *m*/*z* calcd for $C_{15}H_{11}N_5O_3$ [*M*−H]$^-$ 308.0789; found 308.0792 (Δm = +1.0 ppm).

3-(4-(4-Hydroxybenzamido)phenyl)propiolic acid (55)

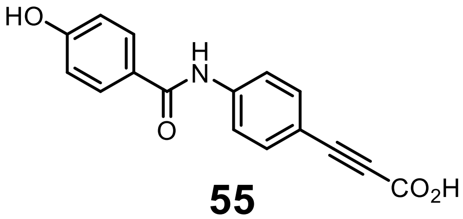

55

BTC (152 mg, 0.514 mmol, 0.45 equiv) was added to a solution of 4-acetoxybenzoic acid (**54**) (309 mg, 1.71 mmol, 1.50 equiv) in dry THF (10 mL) and the solution was cooled down to 0 °C. 2,4,6-collidine (1.82 mL, 13.7 mmol, 12.0 equiv) was added dropwise and the resulting suspension was stirred at that temperature for 45 min. Subsequently, a premixed solution of methyl 3-(4-aminophenyl)propiolate (**53**) (200 mg, 1.14 mmol, 1.00 equiv) and DIPEA (1.99 mL, 11.4 mmol, 10.0 equiv) in dry THF (5 mL) was added dropwise to the suspension and the reaction mixture was stirred for 16 h while warming up to room temperature. After removing all volatiles under reduced pressure, the residue was taken up in EtOAc and washed with 1 N $HCl_{(aq.)}$ (3×), satd. aq. $NaHCO_3$ (3×), and brine. The organic phase was dried over anhyd. Na_2SO_4 and concentrated *in vacuo* to obtain the crude product, which was purified by column chromatography on silica gel (*n*-hexane/EtOAc, 6:1–3:1) to afford methyl 3-(4-(4-acetoxybenzamido)phenyl)propiolate (274 mg, 0.809 mmol, 71%) as colourless solid. ^{1}H NMR (400 MHz, [D$_6$]DMSO): δ=10.59 (s, 1H), 8.03–7.97 (m, 2H), 7.94–7.89 (m, 2H), 7.71–7.64 (m, 2H), 7.34–7.29 (m, 2H), 3.78 (s, 3H), 2.31 (s, 3H). ^{13}C NMR (101 MHz, [D$_6$]DMSO): δ=169.0, 165.2, 153.7, 153.1, 141.9, 133.8, 129.4, 121.9, 120.0, 112.6, 86.6, 80.1, 52.9, 20.9. HRMS (ESI): *m*/*z* calcd for $C_{19}H_{15}NO_5$ [*M*+H]$^+$

338.1023; found 338.1024 (Δm = +0.3 ppm). To a solution of methyl 3-(4-(4-acetoxybenzamido)phenyl)propiolate (260 mg, 771 µmol, 1.00 equiv) in a mixture of THF (7 mL) and MeOH (7 mL) was added 3 N $KOH_{(aq.)}$ (7 mL) and the reaction mixture was stirred at room temperature for 4 h. After removing the organic solvents *in vacuo*, the residue was taken up in EtOAc and washed with 1 N $HCl_{(aq.)}$ (3×) and brine. The organic phase was dried over anhyd. Na_2SO_4 and concentrated under reduced pressure to obtain the title compound **55** (215 mg, 771 µmol, quant.). ^{1}H NMR (400 MHz, [D$_6$]DMSO): δ=10.28 (s, 1H), 10.19 (s, 1H), 7.92–7.82 (m, 4H), 7.64–7.57 (m, 2H), 6.91–6.84 (m, 2H). ^{13}C NMR (101 MHz, [D$_6$]DMSO): δ=165.3, 160.9, 140.2, 132.3, 129.8, 124.9, 119.9, 116.1, 115.0, 48.6. HRMS (ESI): *m*/*z* calcd for $C_{16}H_{11}NO_4$ [*M*+H]$^+$ 282.0761; found 282.0760 (Δm = −0.4 ppm).

Proton and (^{1}H,^{13}C)-HSQC NMR Spectra of Albicidin Derivatives

Compound 3

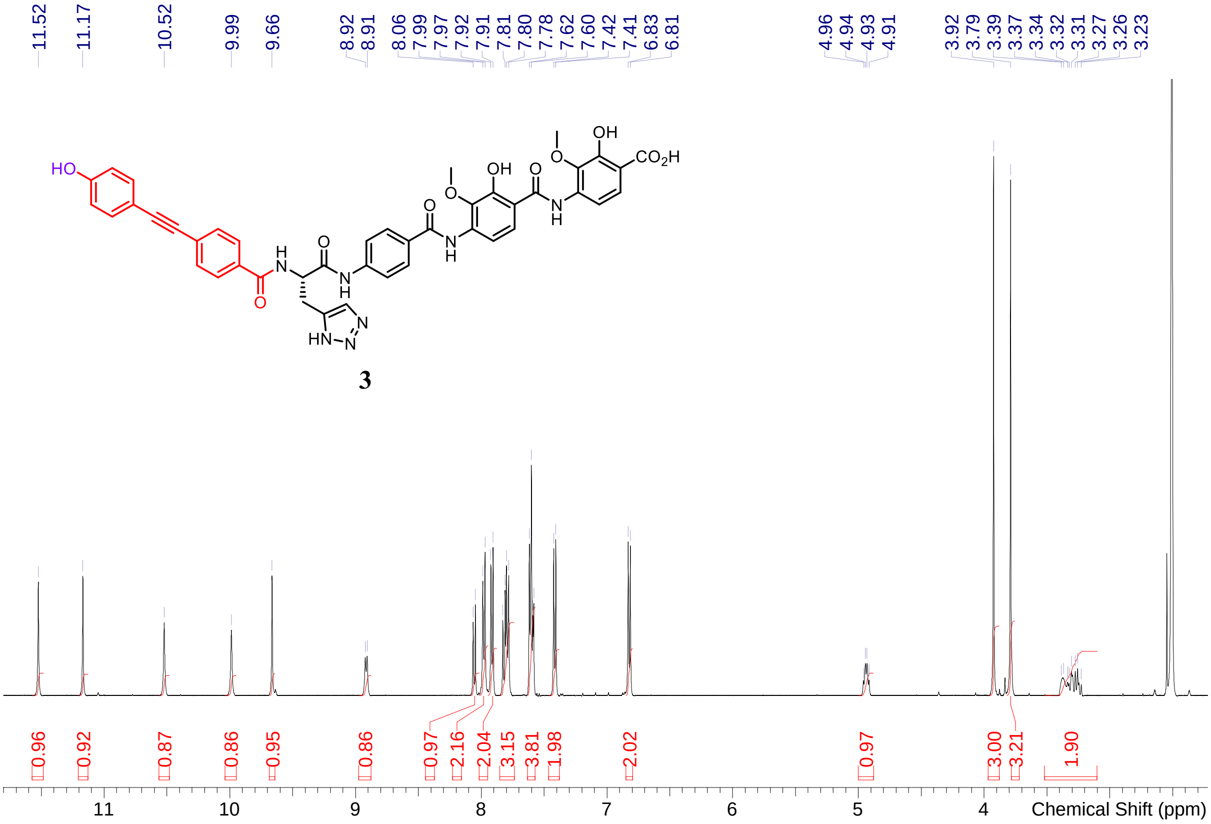

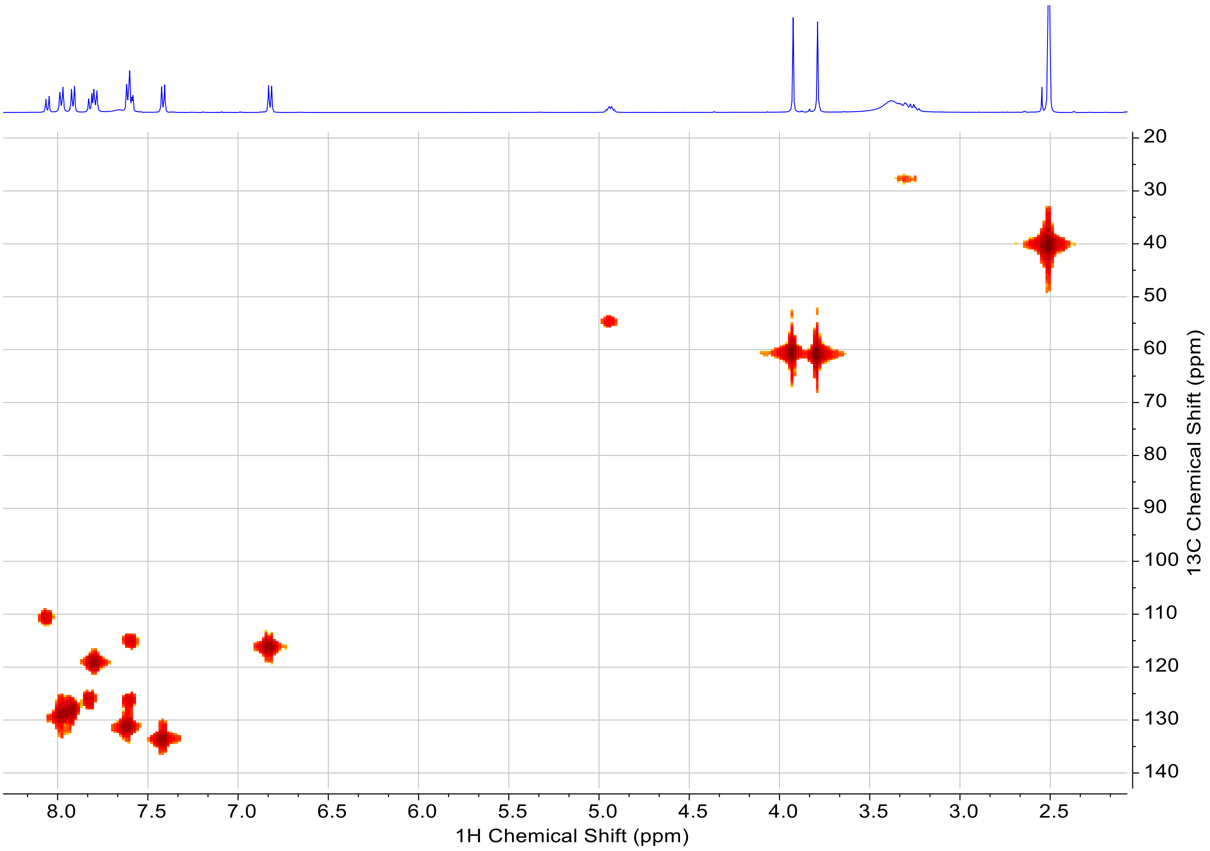

Compound 4

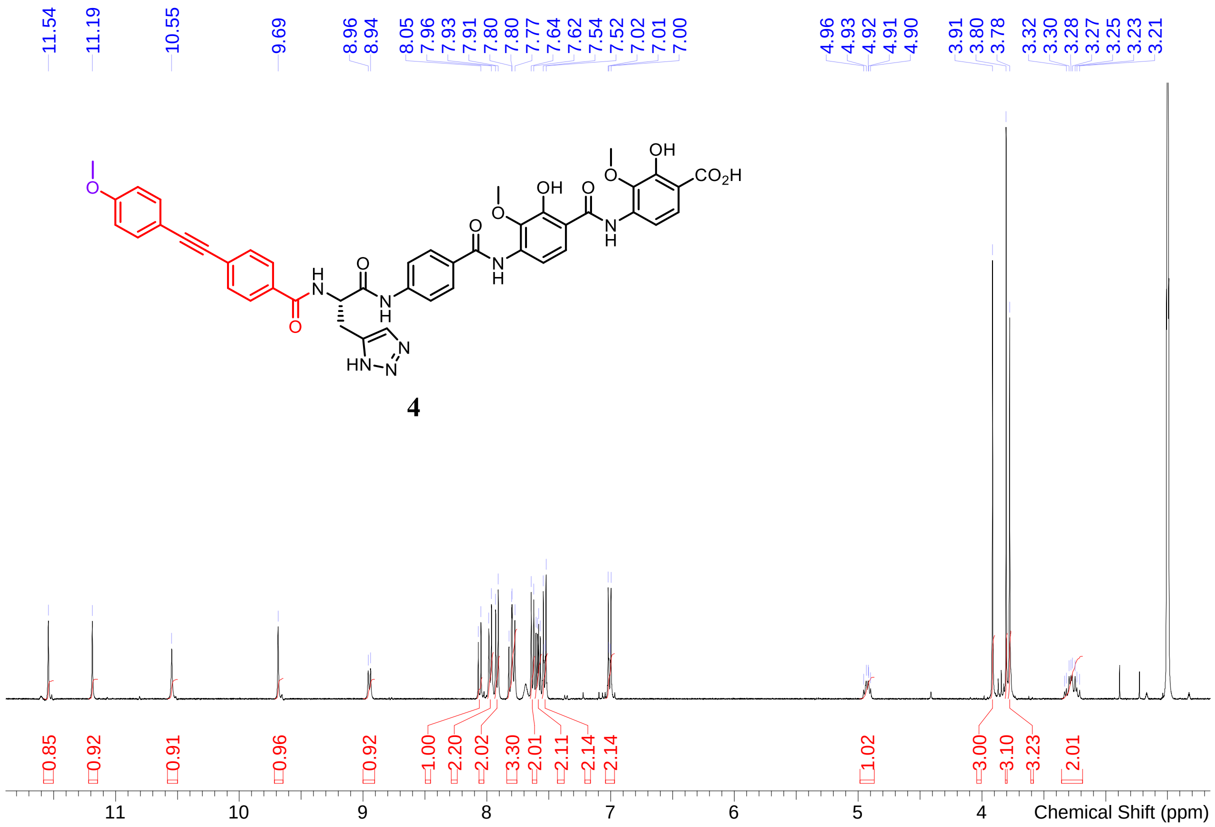

11.54
11.19
10.55
9.69
8.96
8.94
8.05
7.96
7.93
7.91
7.80
7.80
7.77
7.64
7.62
7.54
7.52
7.02
7.01
7.00
4.96
4.93
4.92
4.91
4.90
3.91
3.80
3.78
3.32
3.30
3.28
3.27
3.25
3.23
3.21
OH
CO2H
O
OH
O
O
N
H
O
N
H
H
N
O
N
H
O
HN
N
N
4
0.85
0.92
0.91
0.96
0.92
1.00
2.20
2.02
3.30
2.01
2.11
2.14
2.14
1.02
3.00
3.10
3.23
2.01
11
10
9
8
7
6
5
4
Chemical Shift (ppm)

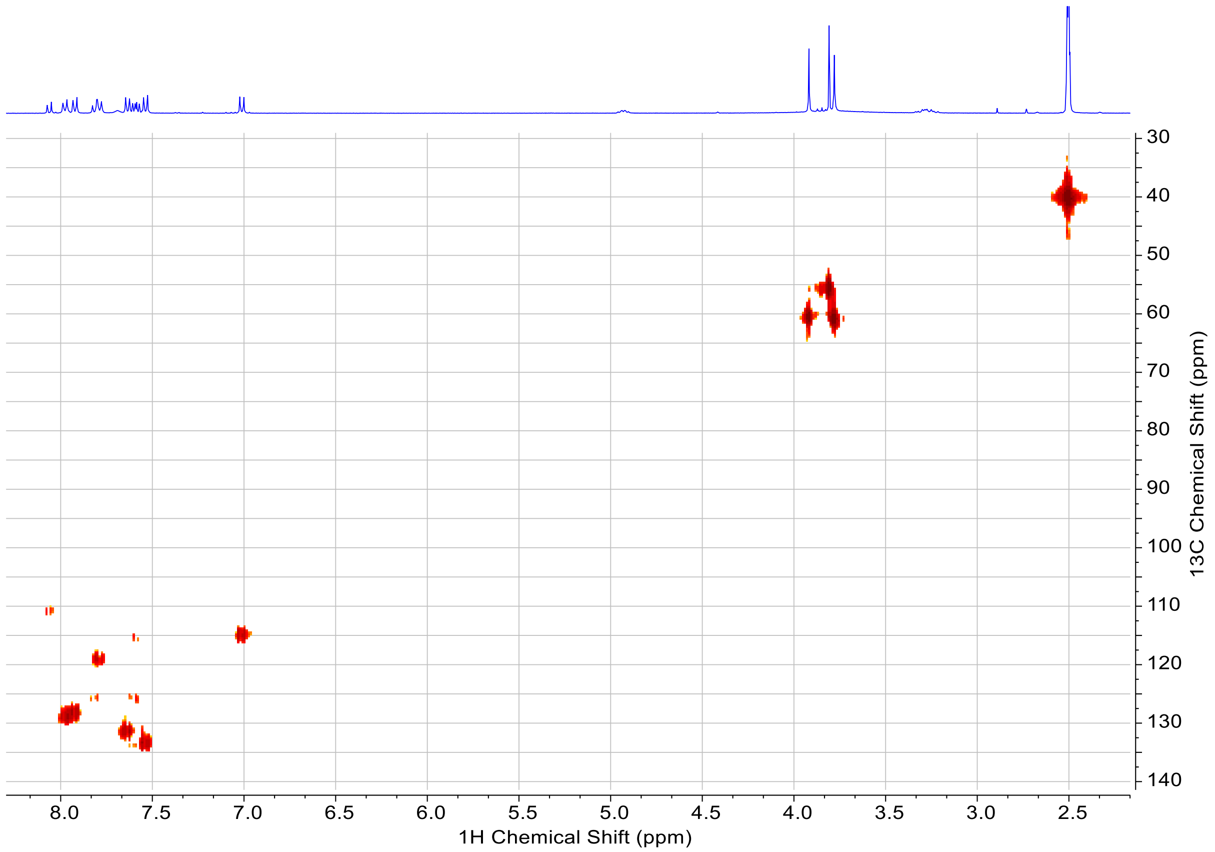

30
40
50
60
70
80
90
100
110
120
130
140
13C Chemical Shift (ppm)
8.0
7.5
7.0
6.5
6.0
5.5
5.0
4.5
4.0
3.5
3.0
2.5
1H Chemical Shift (ppm)

Compound 5

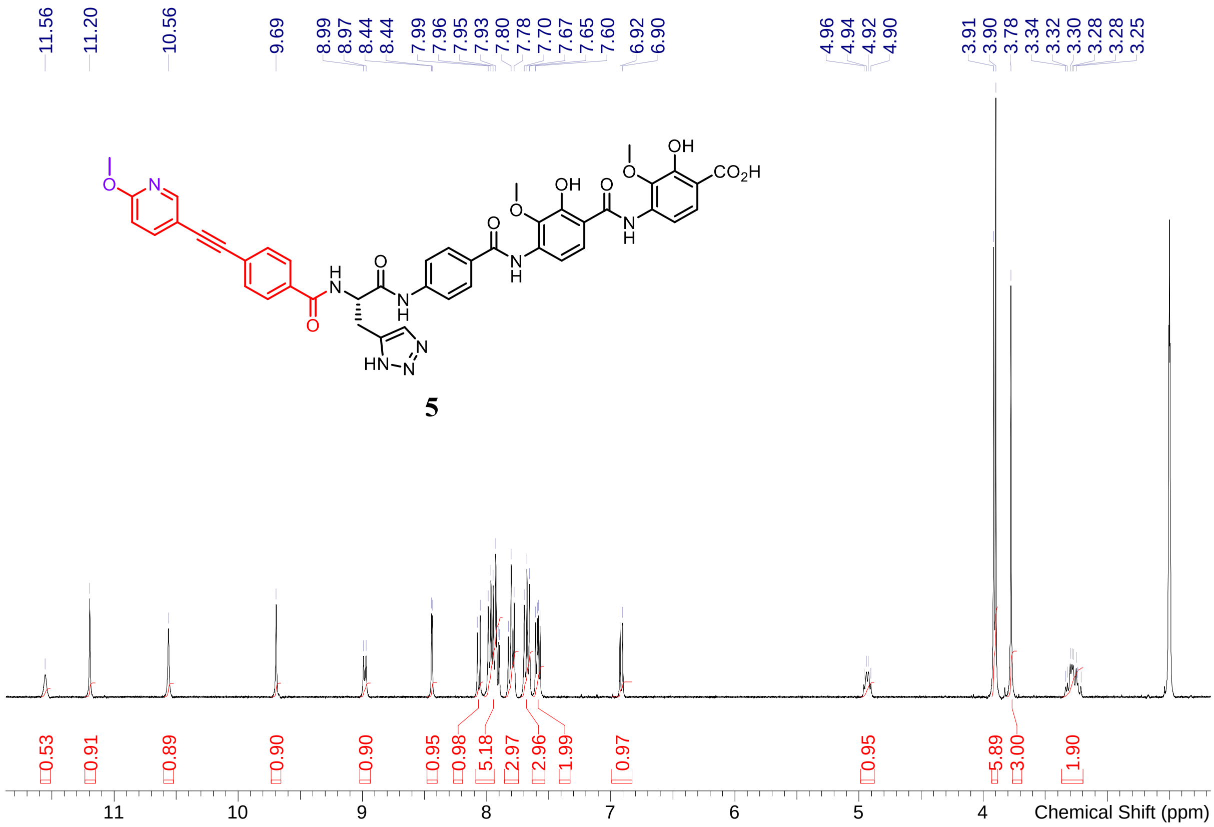

5
Chemical Shift (ppm)

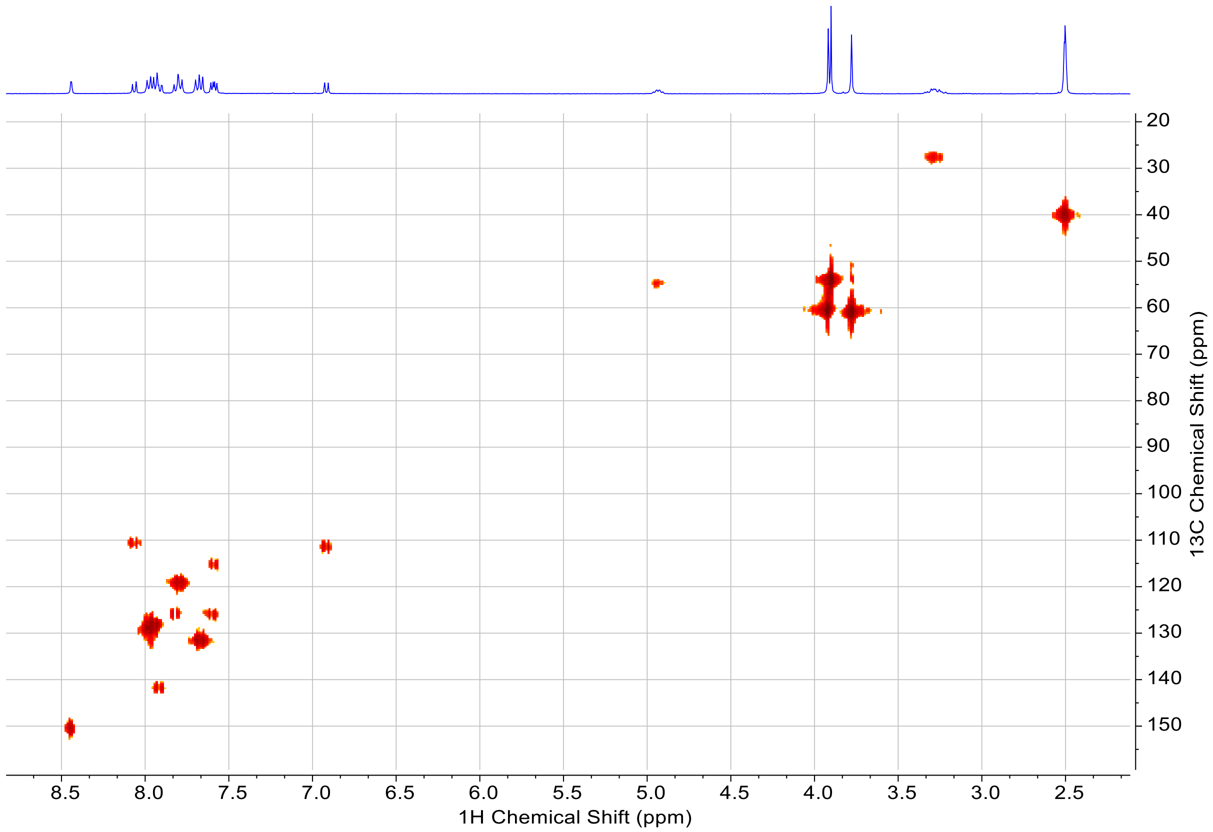

1H Chemical Shift (ppm)
13C Chemical Shift (ppm)

Compound 6

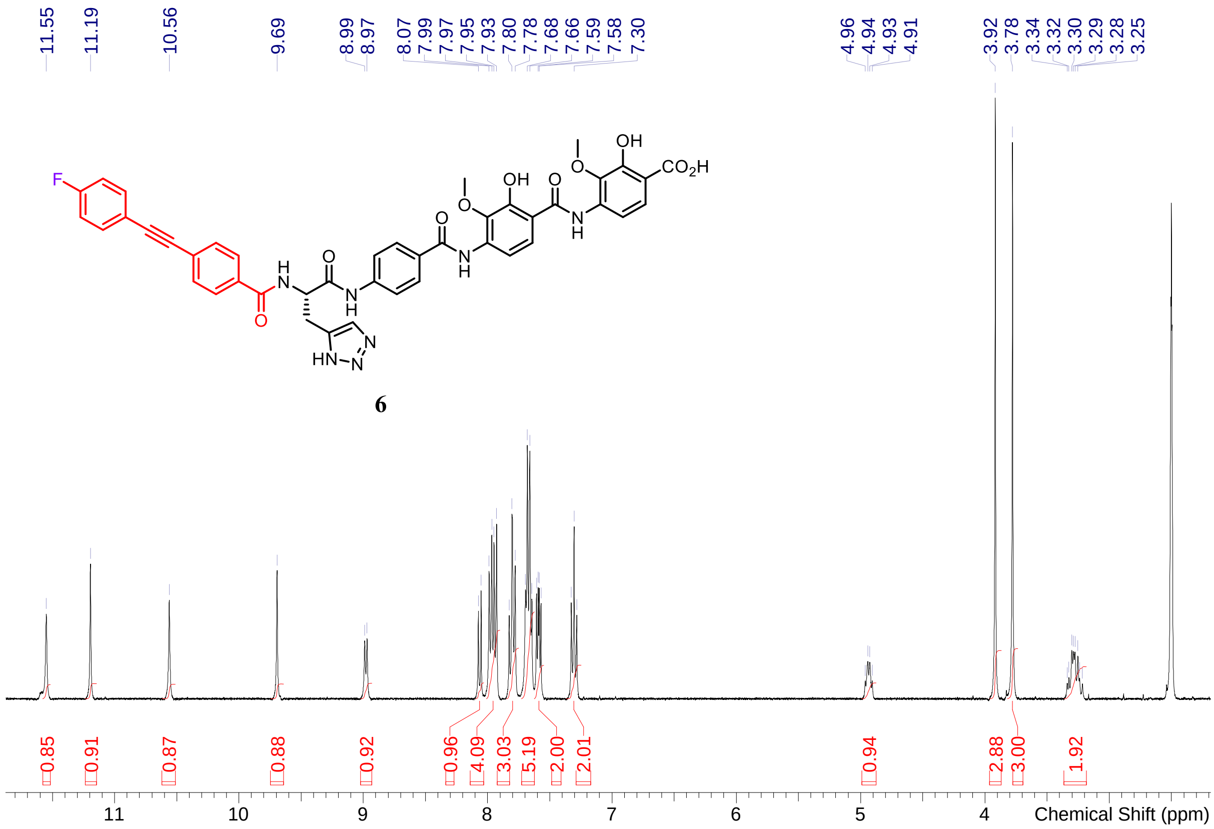
11.55
11.19
10.56
9.69
8.99
8.97
8.07
7.99
7.97
7.95
7.93
7.80
7.78
7.68
7.66
7.59
7.58
7.30
4.96
4.94
4.93
4.91
3.92
3.78
3.34
3.32
3.30
3.29
3.28
3.25
F
OH
O
CO2H
N
H
HN
N
6
0.85
0.91
0.87
0.88
0.92
0.96
4.09
3.03
5.19
2.00
2.01
0.94
2.88
3.00
1.92
11
10
9
8
7
6
5
4
Chemical Shift (ppm)

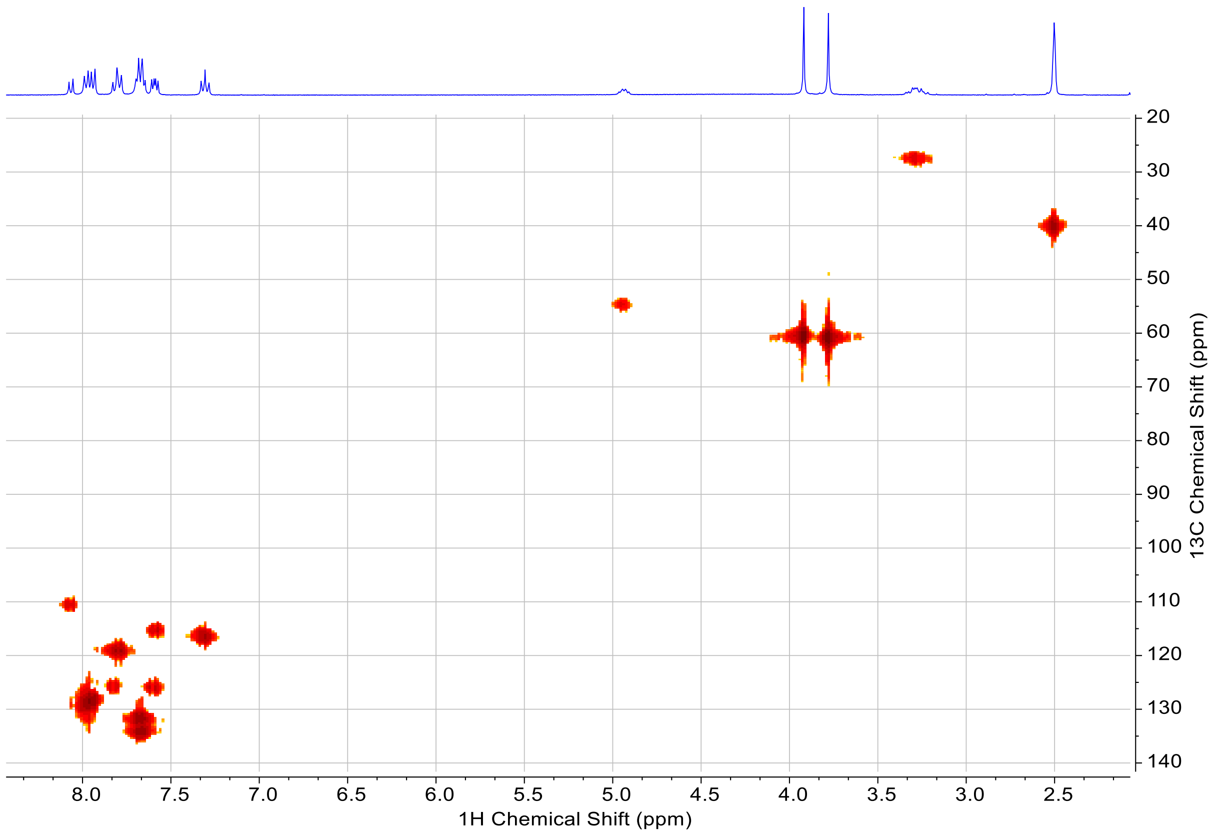
20
30
40
50
60
70
80
90
100
110
120
130
140
13C Chemical Shift (ppm)
8.0
7.5
7.0
6.5
6.0
5.5
5.0
4.5
4.0
3.5
3.0
2.5
1H Chemical Shift (ppm)

Compound 7

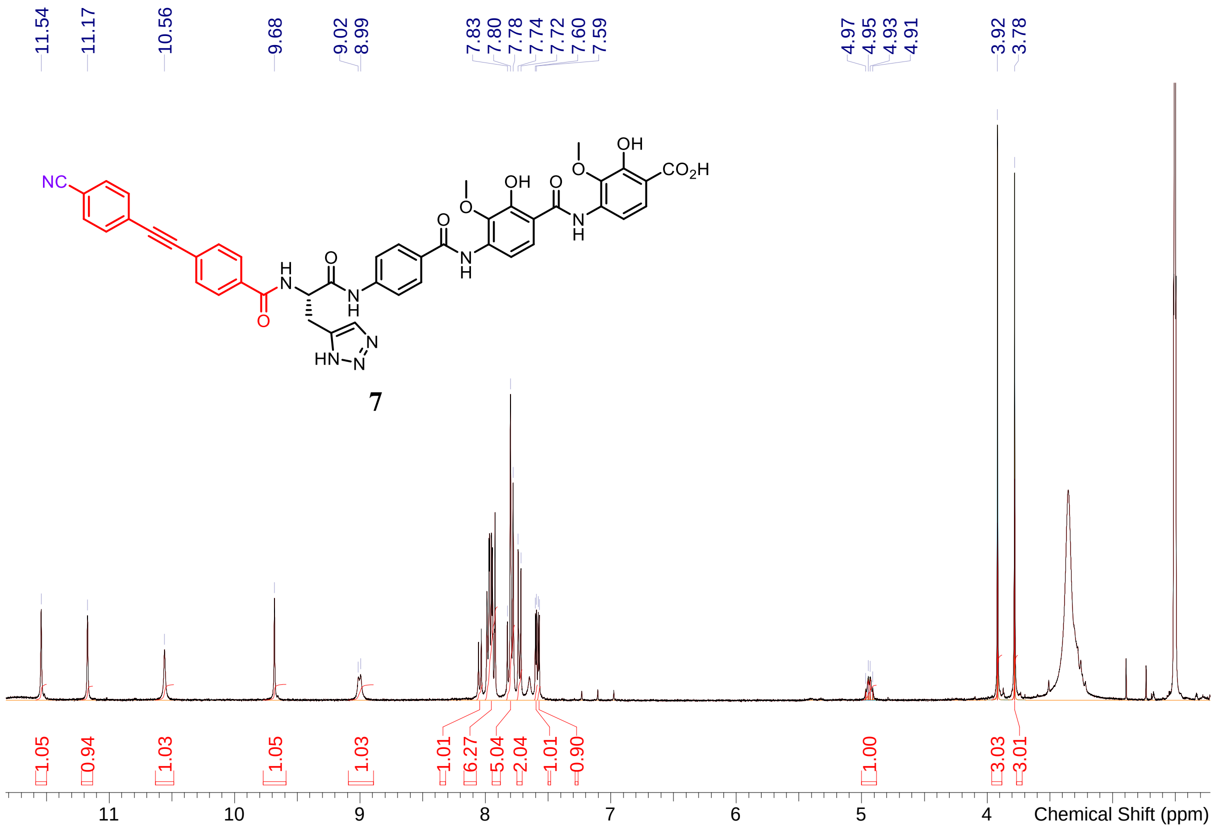
11.54
11.17
10.56
9.68
9.02
8.99
7.83
7.80
7.78
7.74
7.72
7.60
7.59
4.97
4.95
4.93
4.91
3.92
3.78
1.05
0.94
1.03
1.05
1.03
1.01
6.27
5.04
2.04
1.01
0.90
1.00
3.03
3.01
Chemical Shift (ppm)

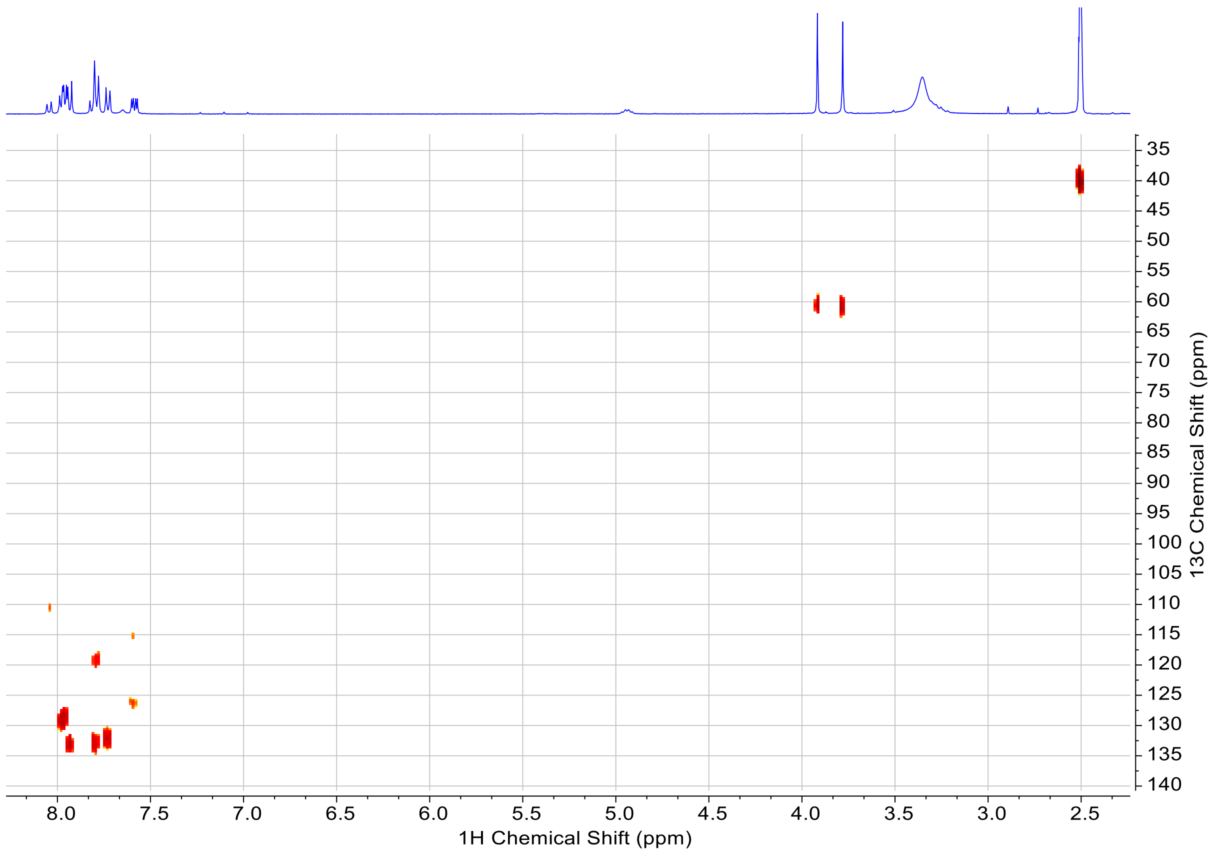
1H Chemical Shift (ppm)
13C Chemical Shift (ppm)

Compound 8

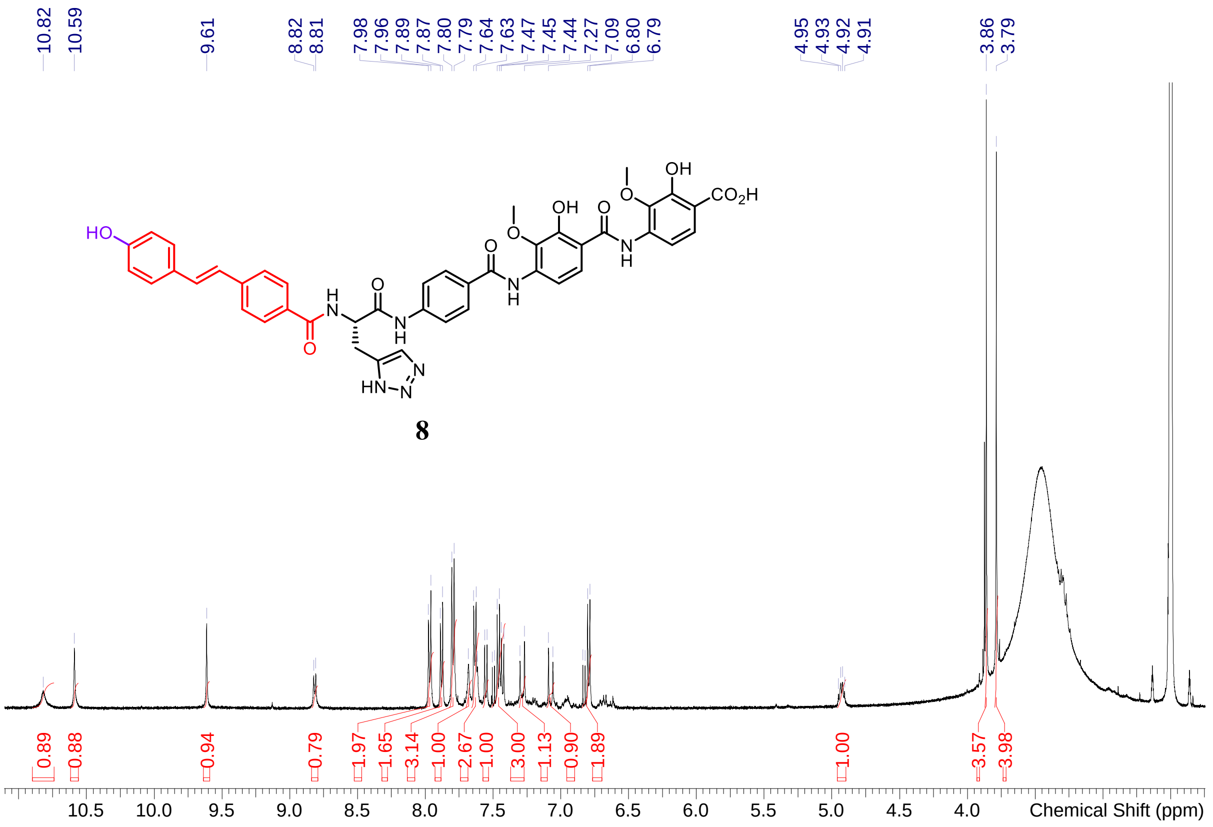
10.82
10.59
9.61
8.82
8.81
7.98
7.96
7.89
7.87
7.80
7.79
7.64
7.63
7.47
7.45
7.44
7.27
7.09
6.80
6.79
4.95
4.93
4.92
4.91
3.86
3.79
HO
H
N
O
N
H
O
N
H
O
OH
O
N
H
O
OH
CO2H
O
HN
N
N
8
0.89
0.88
0.94
0.79
1.97
1.65
3.14
1.00
2.67
1.00
3.00
1.13
0.90
1.89
1.00
3.57
3.98
10.5
10.0
9.5
9.0
8.5
8.0
7.5
7.0
6.5
6.0
5.5
5.0
4.5
4.0
Chemical Shift (ppm)

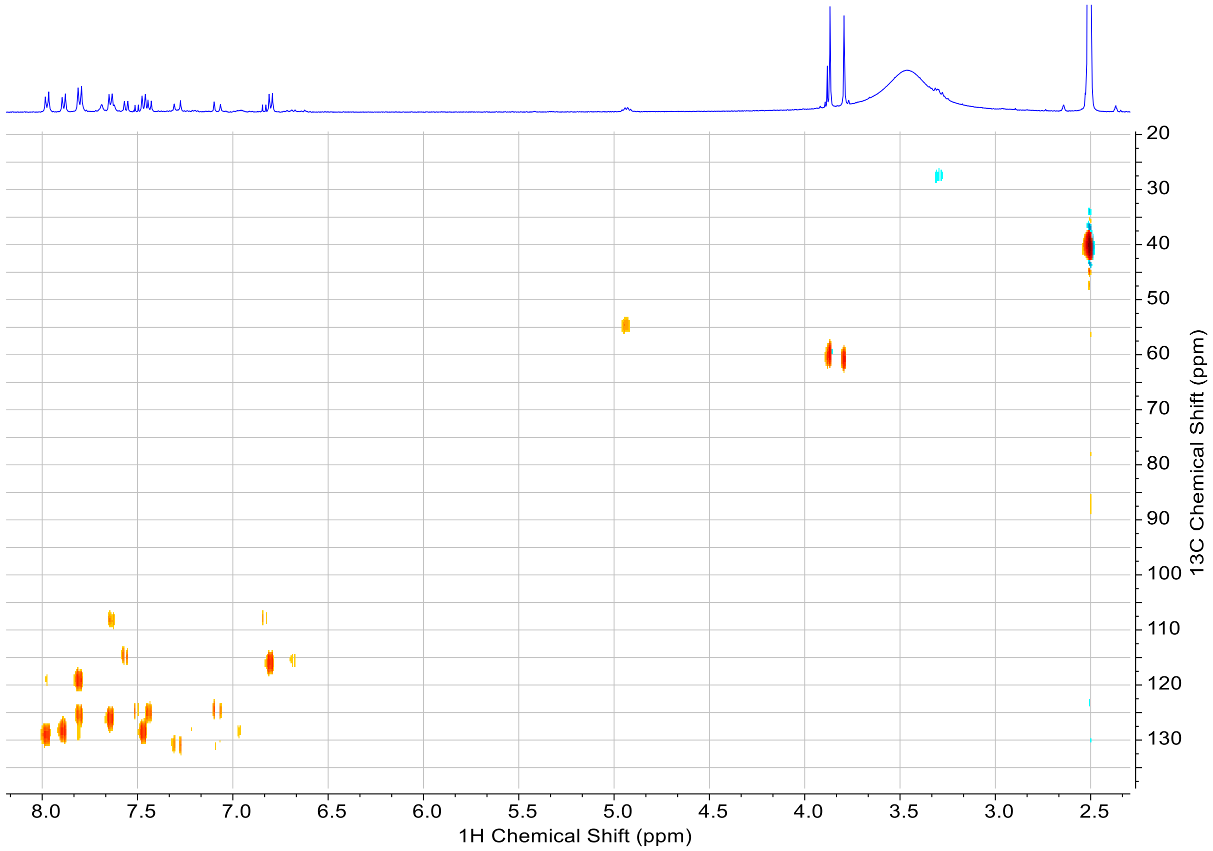
1H Chemical Shift (ppm)
13C Chemical Shift (ppm)
8.0
7.5
7.0
6.5
6.0
5.5
5.0
4.5
4.0
3.5
3.0
2.5
20
30
40
50
60
70
80
90
100
110
120
130

Compound 9

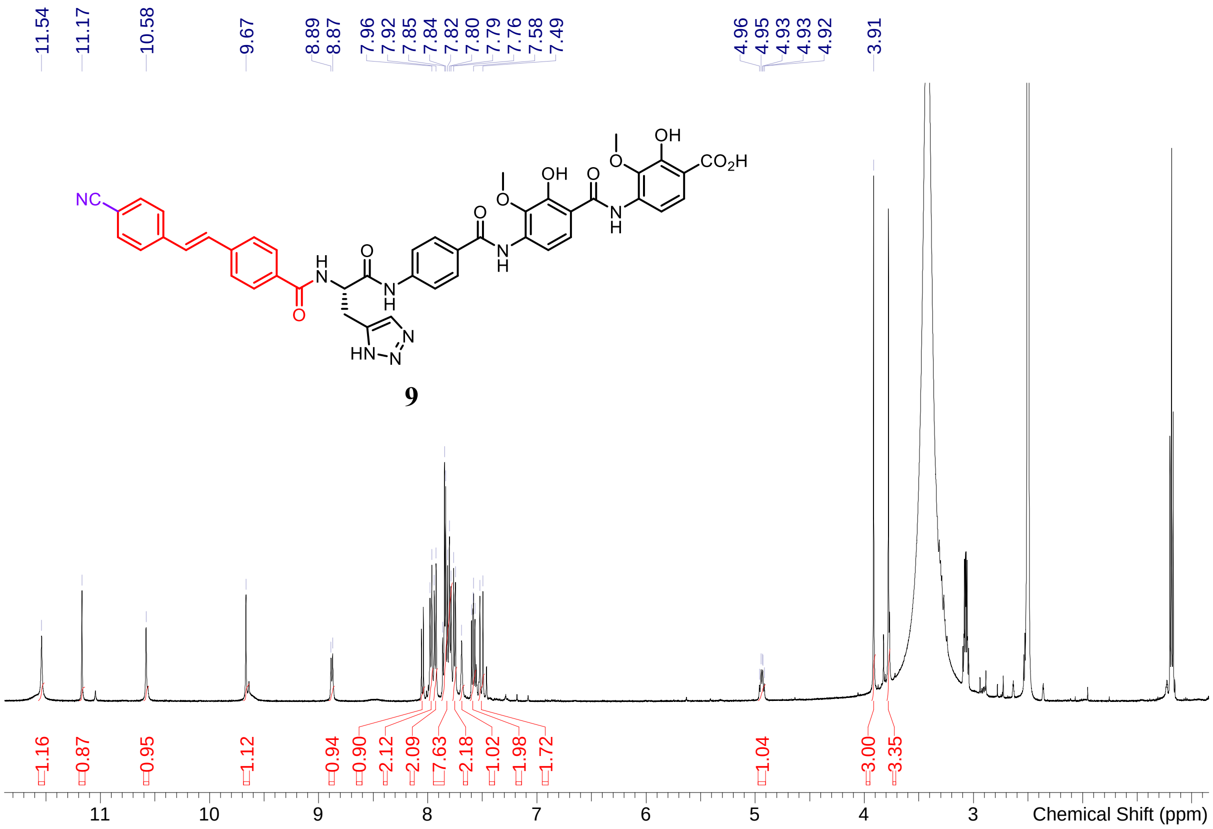
11.54
11.17
10.58
9.67
8.89
8.87
7.96
7.92
7.85
7.84
7.82
7.80
7.79
7.76
7.58
7.49
4.96
4.95
4.93
4.93
4.92
3.91
NC
OH
CO_2H
O
N
H
HN
9
1.16
0.87
0.95
1.12
0.94
0.90
2.12
2.09
7.63
2.18
1.02
1.98
1.72
1.04
3.00
3.35
Chemical Shift (ppm)

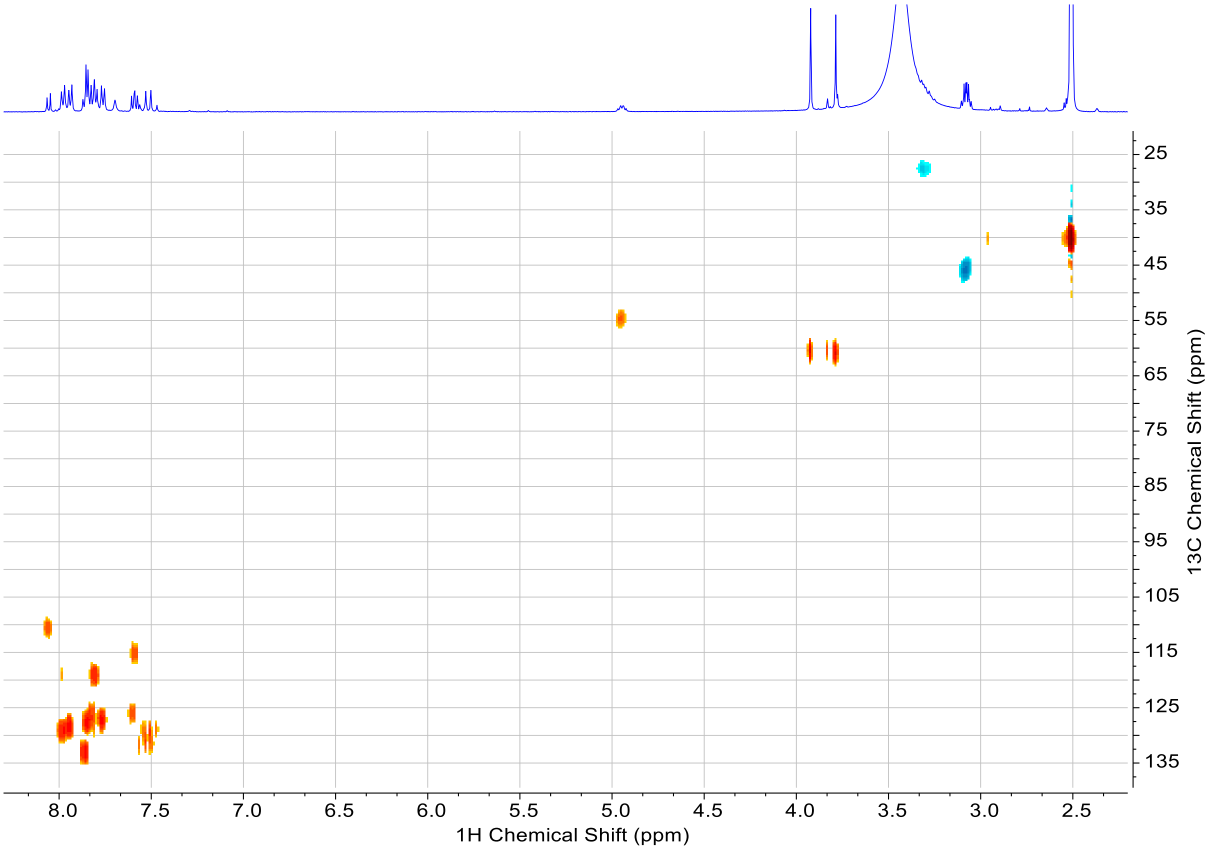
1H Chemical Shift (ppm)
13C Chemical Shift (ppm)

Compound 10

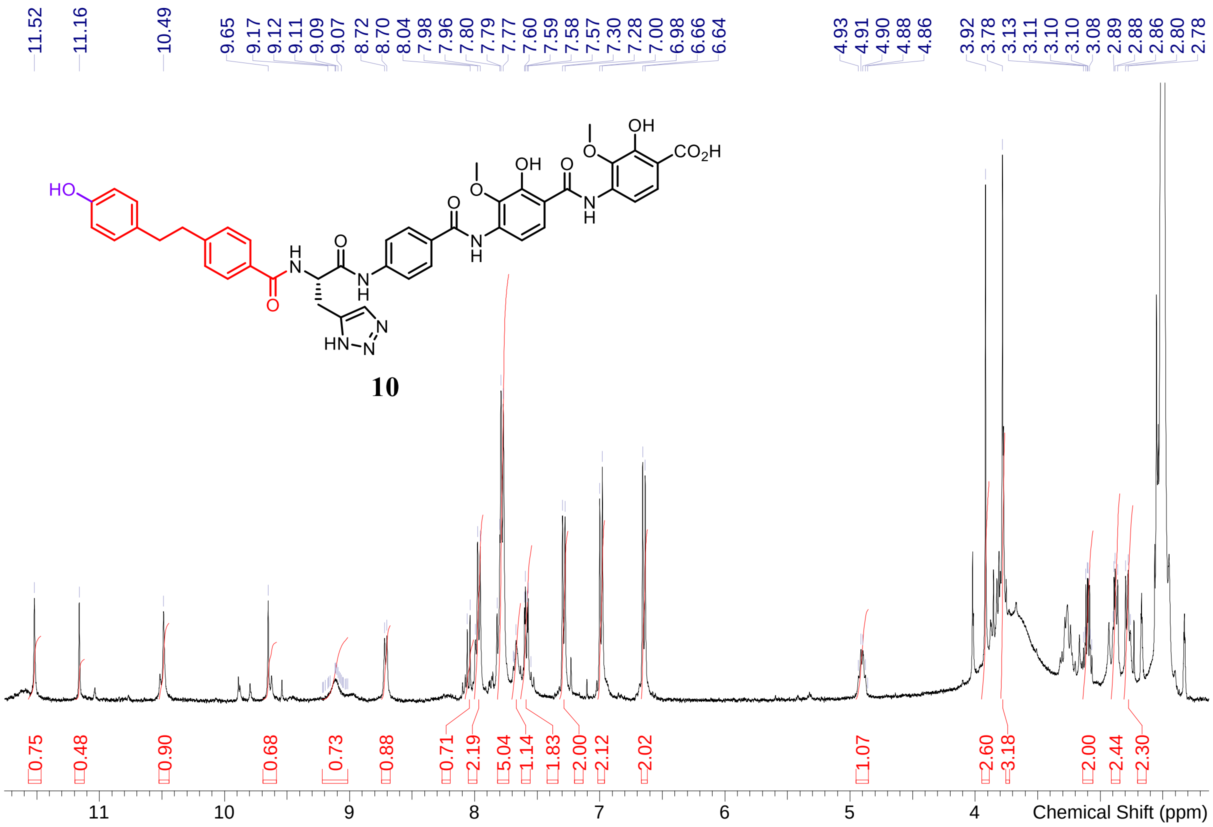

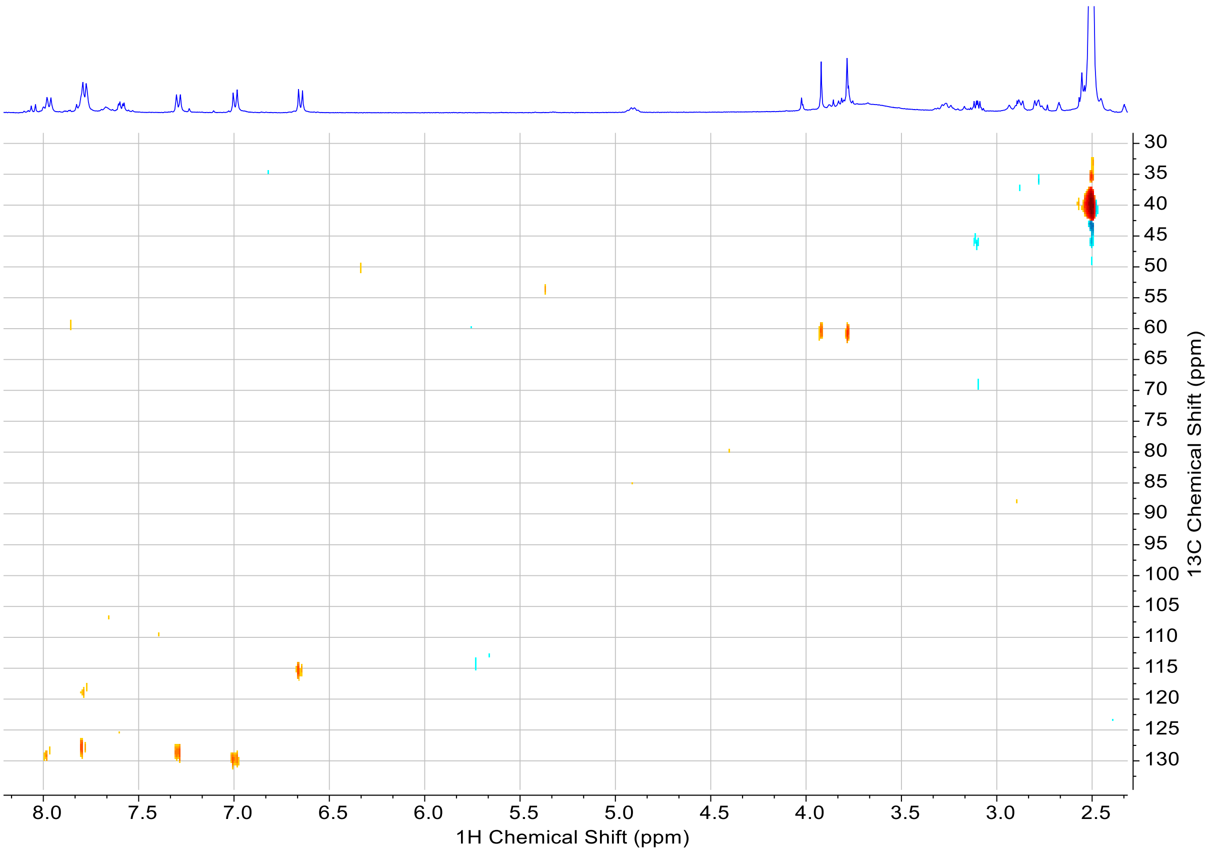

Compound 11

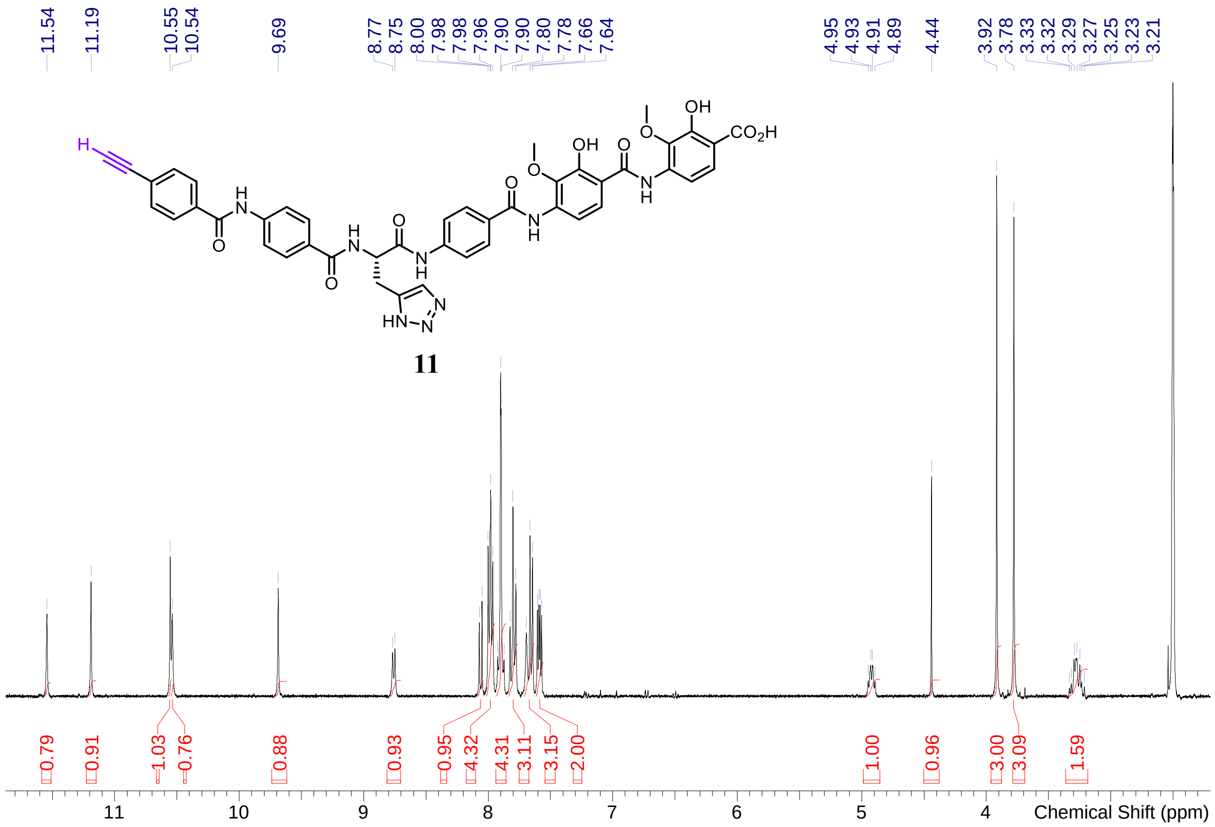
11.54
11.19
10.55
10.54
9.69
8.77
8.75
8.00
7.98
7.98
7.96
7.90
7.90
7.80
7.78
7.66
7.64
4.95
4.93
4.91
4.89
4.44
3.92
3.78
3.33
3.32
3.29
3.27
3.25
3.23
3.21
11
0.79
0.91
1.03
0.76
0.88
0.93
0.95
4.32
4.31
3.11
3.15
2.00
1.00
0.96
3.00
3.09
1.59
Chemical Shift (ppm)

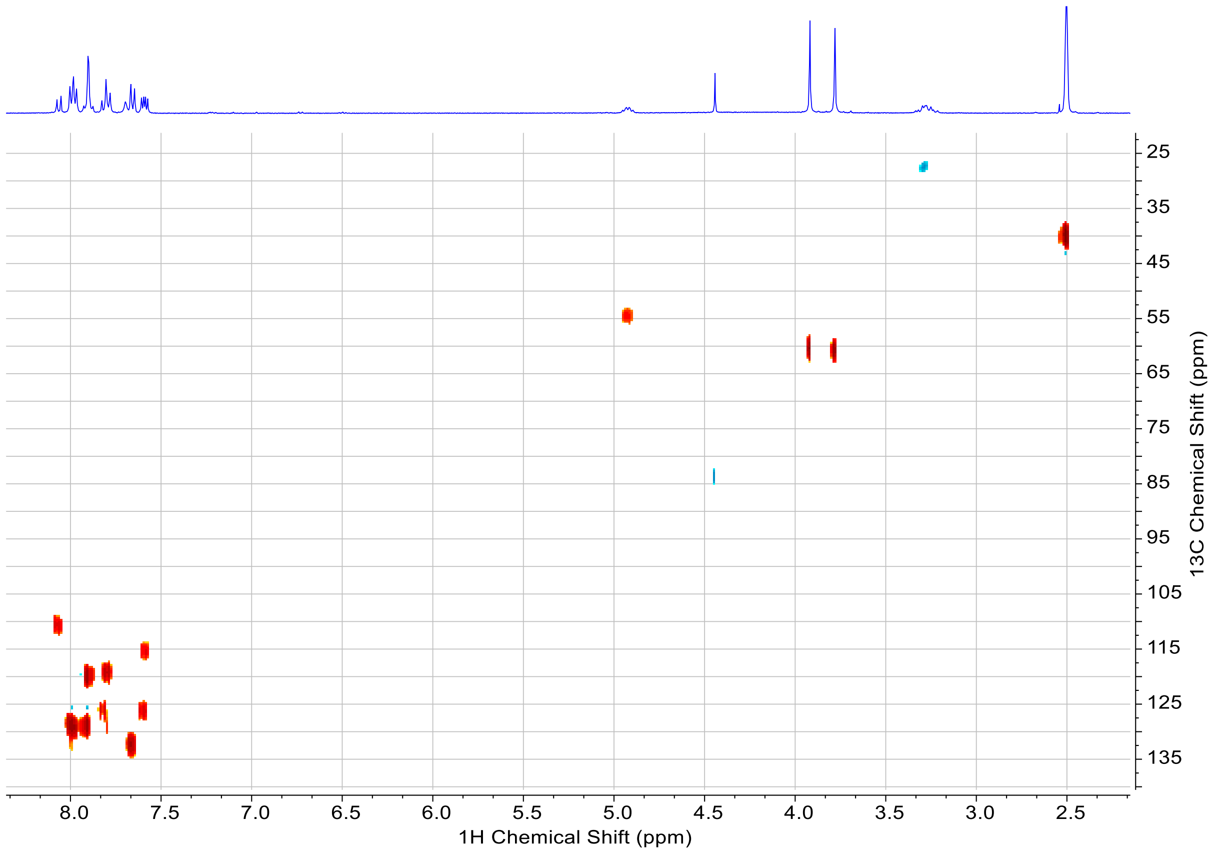
13C Chemical Shift (ppm)
1H Chemical Shift (ppm)

Compound 12

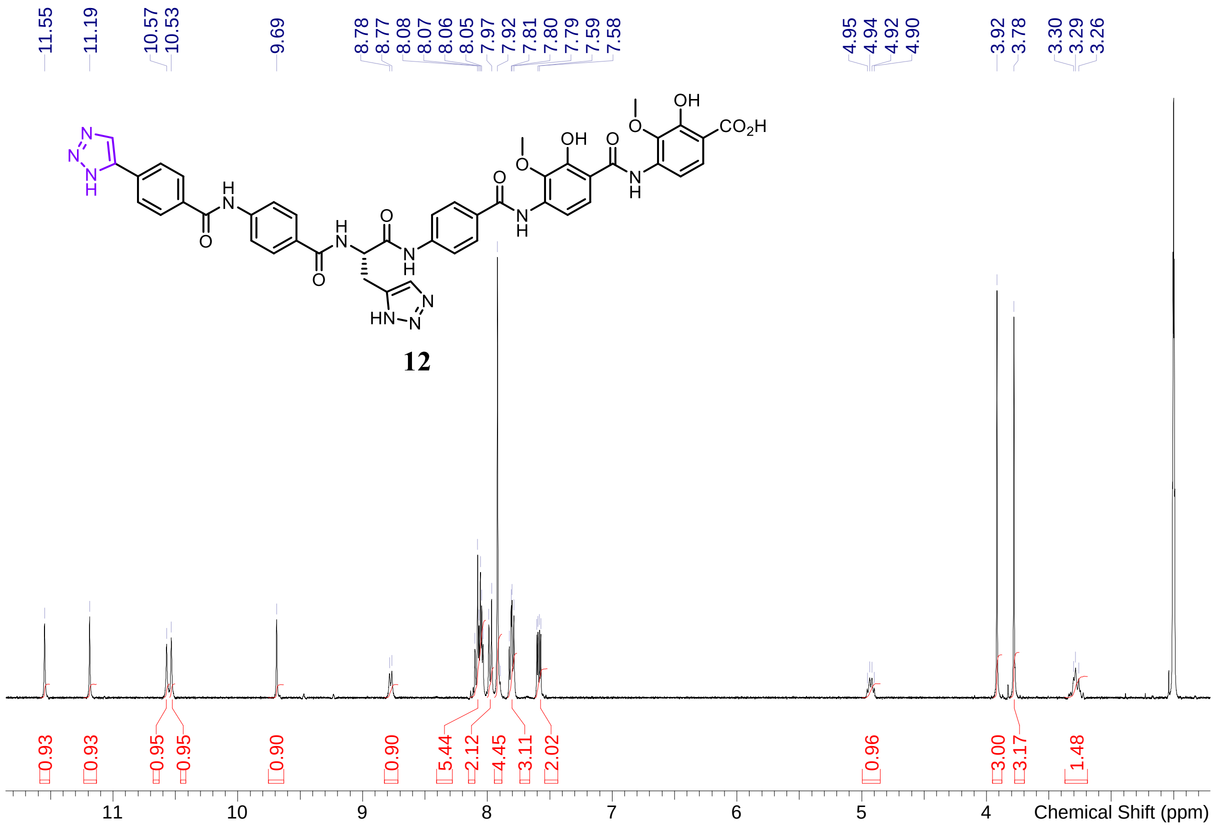

11.55
11.19
10.57
10.53
9.69
8.78
8.77
8.08
8.07
8.06
8.05
7.97
7.92
7.81
7.80
7.79
7.59
7.58
4.95
4.94
4.92
4.90
3.92
3.78
3.30
3.29
3.26
OH
CO_2H
12
0.93
0.93
0.95
0.95
0.90
0.90
5.44
2.12
4.45
3.11
2.02
0.96
3.00
3.17
1.48
Chemical Shift (ppm)

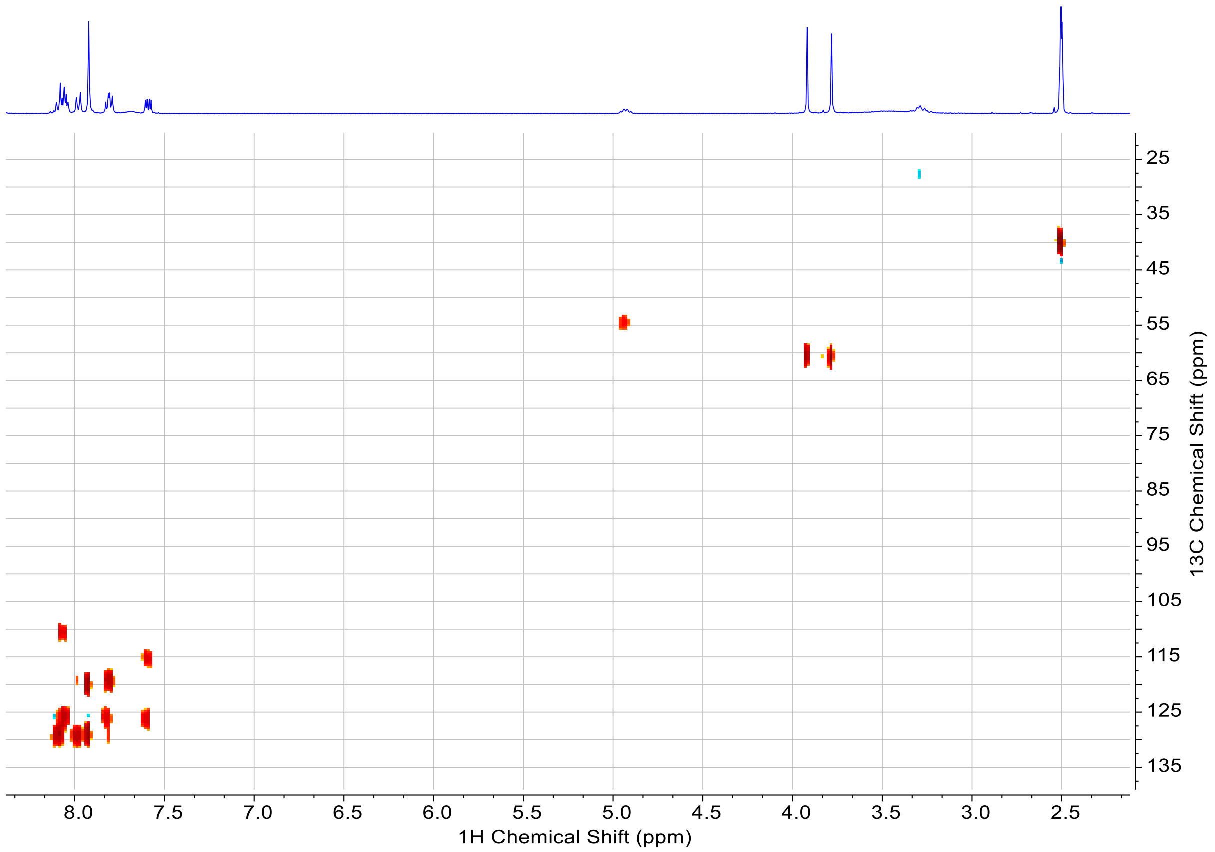

1H Chemical Shift (ppm)
13C Chemical Shift (ppm)

Compound 13

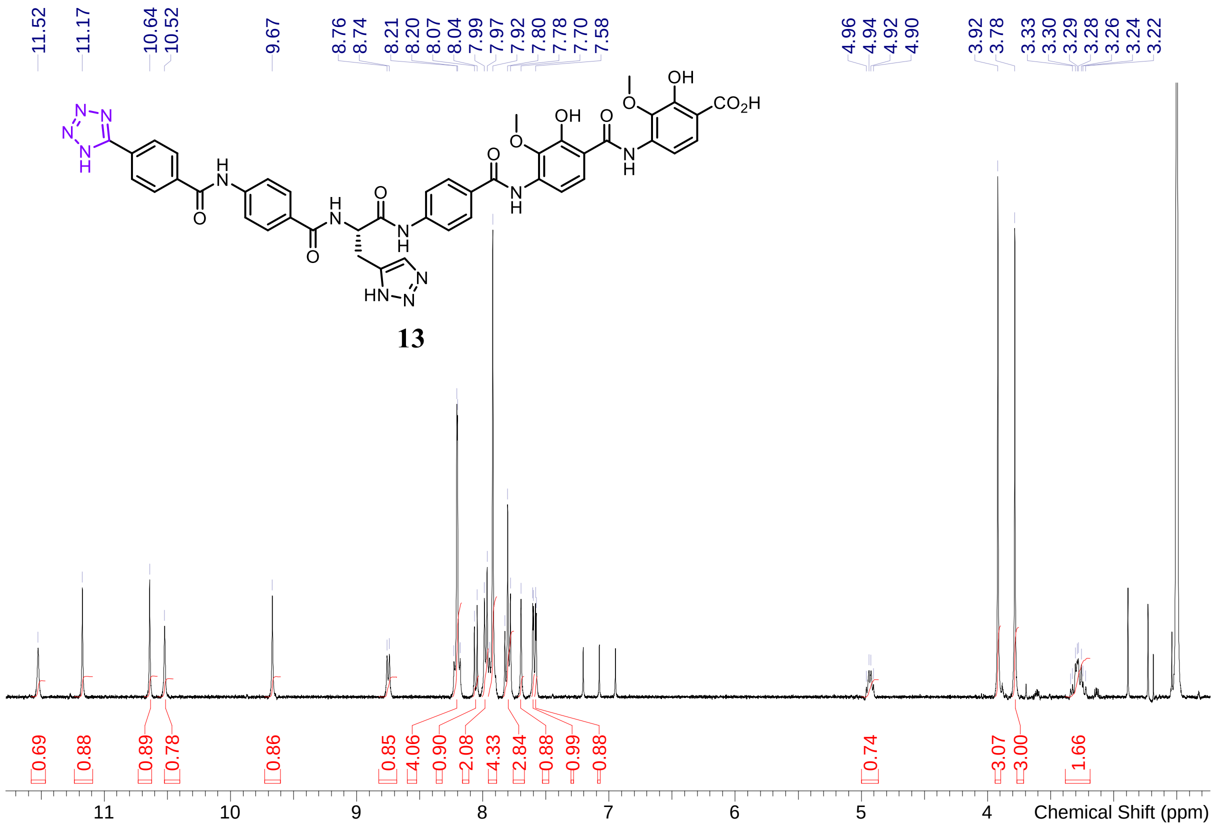

13
Chemical Shift (ppm)

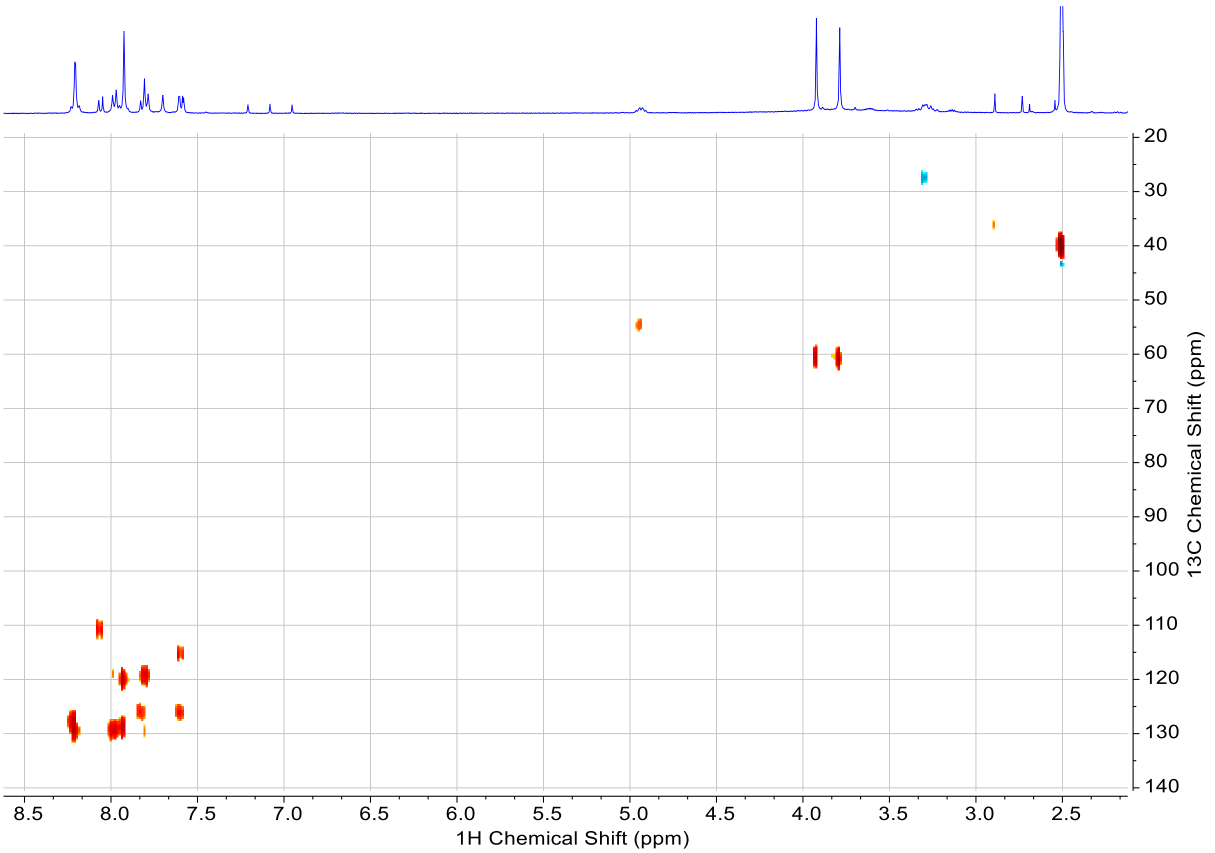

1H Chemical Shift (ppm)
13C Chemical Shift (ppm)

Compound 14

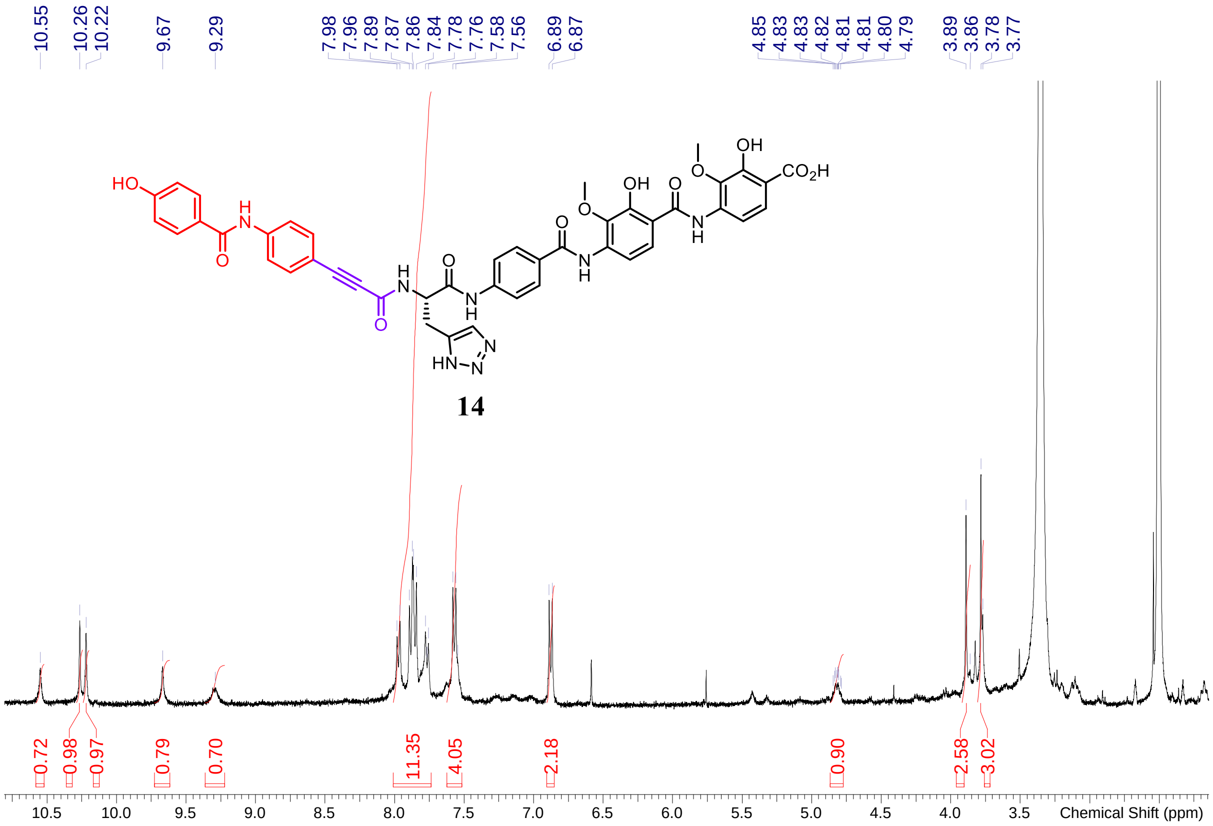

Compound 15

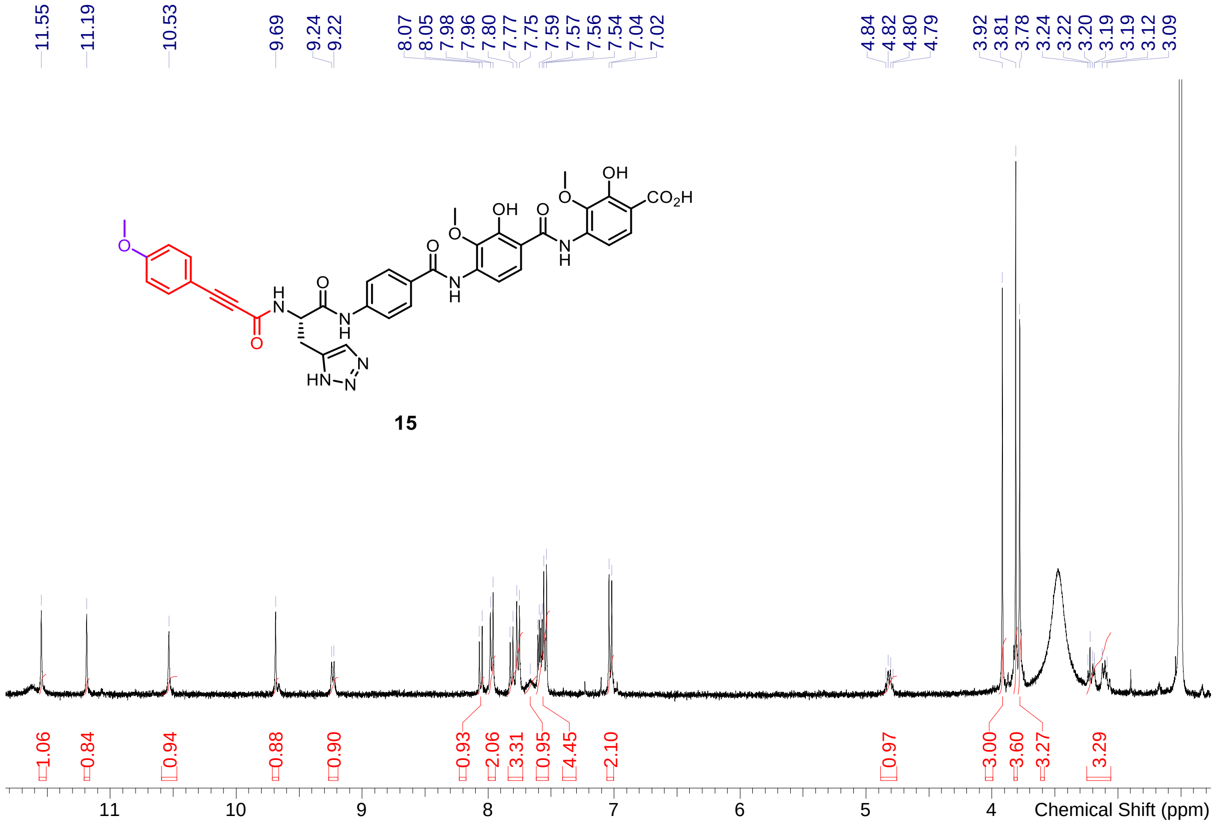

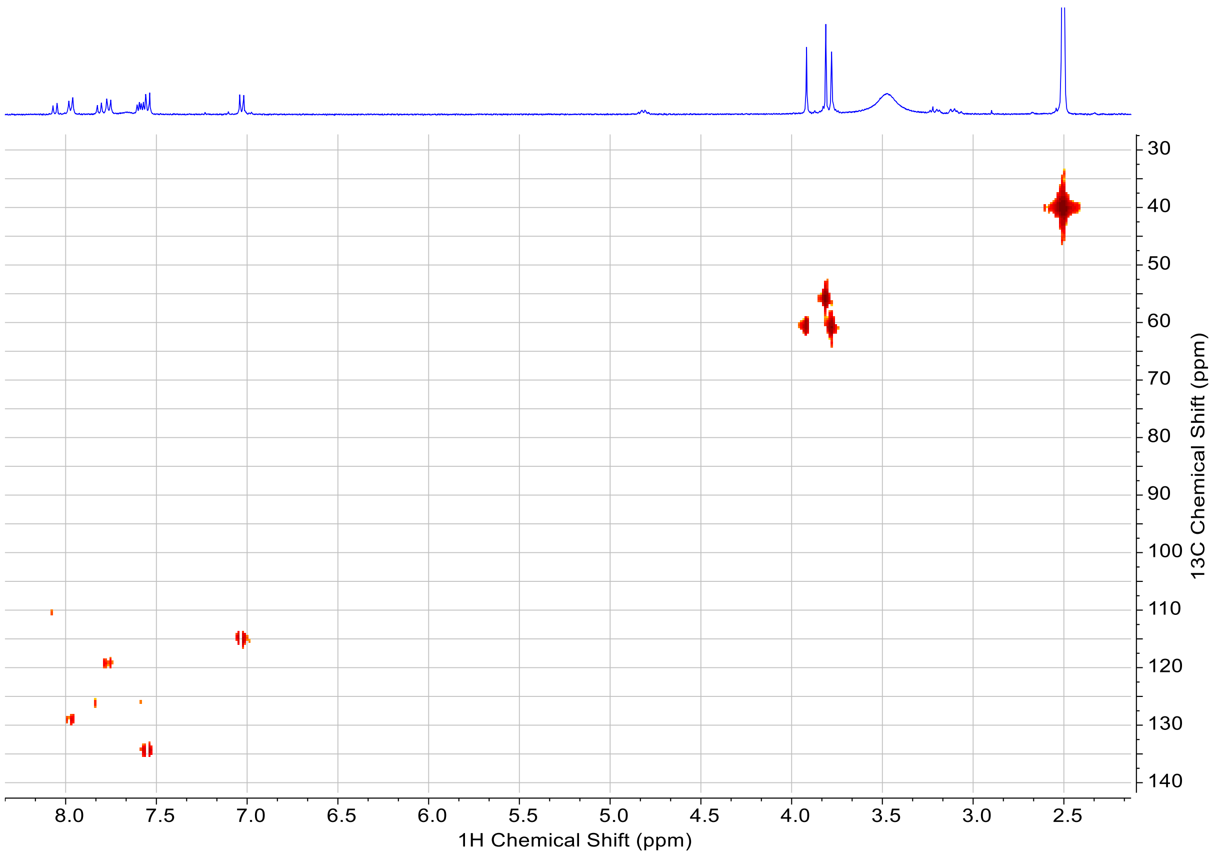
30
40
50
60
70
80
90
100
110
120
130
140
13C Chemical Shift (ppm)
8.0
7.5
7.0
6.5
6.0
5.5
5.0
4.5
4.0
3.5
3.0
2.5
1H Chemical Shift (ppm)

References

[1] M. P. Weinstein, *Methods for dilution antimicrobial susceptibility tests for bacteria that grow aerobically – ninth edition*. M07-A10/Vol. 38 No.2; National Committee for Clinical Laboratory Standards, Villanova, Pennsylvania, **2012**.

Author Contributions

I. Behroz, L. Kleebauer, K. Hommernick, M. Seidel, S. Grätz, A. Mainz, J. B. Weston, and R. D. Süssmuth designed the experiments. L. Kleebauer prepared two compounds and provided precursors. K. Hommernick prepared one compound and provided precursors. I. Behroz prepared the remaining compounds and precursors. M. S. performed the MIC and gyrase inhibition assays. S. Grätz and A. Mainz performed the NMR experiments regarding the photoisomeriszation of albicidin. I. Behroz, L. Kleebauer, K. Hommernick, A. Mainz, J. B. Weston, and R. D. Süssmuth wrote the manuscript. All authors read, discussed, and approved the manuscript.
